# Deciphering the Role of Inorganic Nanoparticles’ Surface Functionalization on Biohybrid Microbial Photoelectrodes

**DOI:** 10.1021/acsami.4c12070

**Published:** 2024-10-20

**Authors:** Pierluigi Lasala, Rosa Maria Matteucci, Saverio Roberto Volpicella, Jefferson Honorio Franco, Doriana Debellis, Federico Catalano, Antonella Milella, Roberto Grisorio, Gian Paolo Suranna, Angela Agostiano, Maria Lucia Curri, Elisabetta Fanizza, Matteo Grattieri

**Affiliations:** † Department of Chemistry, University of Bari, Via Orabona 4, Bari 70125, Italy; ‡ CNR-IPCF, SS Bari, Via Orabona 4, Bari 70125, Italy; § Polytechnic University of Bari, Via Orabona 4, Bari 70125, Italy; ∥ Electron Microscopy Facility, Istituto Italiano di Tecnologia, Via Morego, 30, Genoa 16163, Italy; ⊥ Dipartimento di Ingegneria Civile, Ambientale, del Territorio, Edile e di Chimica (DICATECh), Politecnico di Bari, Via Orabona 4, Bari 70125, Italy; # Consorzio Interuniversitario Nazionale per la Scienza e Tecnologia dei Materiali (INSTM), Bari Research Unit, Via Orabona 4, Bari 70125, Italy; 7 CNR-NANOTEC, Institute of Nanotechnology, c/o Campus Ecoteckne, Via Monteroni, Lecce 73100, Italy

**Keywords:** inorganic nanoparticles, biohybrid systems, photobioelectrochemistry, surface functionalization, whole cell-electrode, current generation

## Abstract

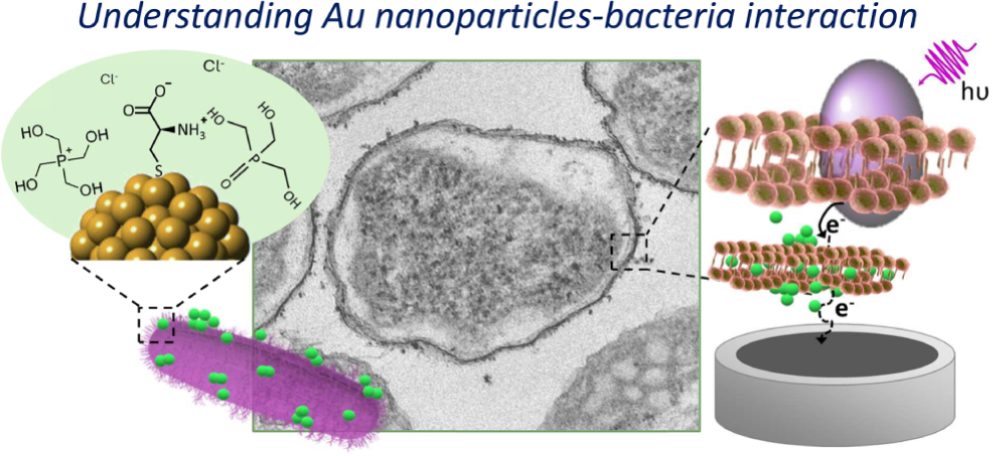

Shedding light on the interaction between inorganic nanoparticles (NPs) and living microorganisms is at the basis of the development of biohybrid technologies with improved performance. Au NPs have been shown to be able to improve the extracellular electron transfer (EET) in intact bacterial cells interfaced with an electrode; however, detailed information on the role of NP-surface properties in their interaction with bacterial membranes is still lacking. Herein, we unveil how the surface functionalization of Au NPs influences their interaction with photosynthetic bacteria, focusing on cell morphology, growth kinetics, NPs localization, and electrocatalytic performance. We show that functionalization of Au NPs with cysteine in the zwitterionic form results in a uniform NPs distribution in purple bacteria, specifically locating the NPs within the outer-membrane/periplasmic space of bacterial cells. These biohybrid cells, when coupled with an electrode, exhibit enhanced EET and increased (photo)current generation, paving the way for the future development of rationally designed biohybrid electrochemical systems.

## Introduction

The use of intact microbial cells as biocatalysts for the development of bioelectrochemical systems has found several applications in recent years,^1^ going from localized power generation^2^ to (self-powered) biosensing^3−6^ and bioelectrosynthesis of high-value products.^7−9^ Despite these promising applications, a critical limitation to the implementation of such technologies remains the hindered extracellular electron transfer (EET) process, which results in low current generation and limited product yields. Various methodologies have been reported to enhance EET such as the rational design of artificial redox mediation systems,^10,11^ use of bioinspired approaches,^12^ and synthetic biology for engineering heterologous electron transfer pathways.^13,14^ Recently, the integration of inorganic metal,^15^ semiconductor^16^ or metal oxide nanoparticles^17^ (NPs), and graphitic carbon dots^18^ in intact bacterial cells has attracted particular interest for obtaining biohybrids with improved EET. These biohybrid systems combine the merits of nanosized materials with those of the whole bacterial cell.^17^ Notably, silver NPs with *Shewanella oneidensis* cells allowed reaching the maximum power production in microbial fuel cells (0.66 ± 0.03 mW cm^–2^), with a Coulombic efficiency of 81%.^15^

Gold NPs (Au NPs) have been employed to enhance EET in microbial electrodes with oxygenic photosynthetic organisms, such as the cyanobacterium *Synechocystis* sp. PCC 6803.^19^ This application is crucial for improving photomicrobial fuel cells or biophotovoltaics. The interaction of Au NPs with photosynthetic bacteria is particularly advantageous as it not only boosts the photoelectrocatalytic response by improving the EET at the biotic–abiotic interface, but also enhances the solar light harvesting via an antenna-induced mechanism,^20^ and contributes to increased cell metabolisms.^21^

Despite the proven efficacy of NPs in facilitating charge transfer at the cell–electrode interface, the mechanisms regulating NP–bacterium interactions and the localization of the NPs within the bacterial cells have remained underexplored. Current understanding of bacteria–NPs interaction in biohybrid electrodes is based on the fact that NP-modified microbial cells described in literature generally originate from biohybrid systems obtained by *in situ* biosynthesis of NPs using bacterial cells as bionanofactories.^16,22^ Little is known about the detailed mechanisms taking place at the nanomaterial–microbe interface during this process. Endogenous biological agents reduce Au ions during microbial synthesis of Au NPs but the resulting NPs may localize extracellularly, intracellularly, or in the periplasm, depending on the bacterial strains. Recently, synthesis of Au NPs in the intermembrane space of *E. coli* has been developed by leveraging the electron transport chain to enhance microbial energy metabolism.^1^ It should be underlined that the biosynthesis processes face challenges, such as the sensitivity of Au ions precursors to the environment, the susceptibility of bacteria growth to such ions,^22^ the low NP production yield, and the poorly controlled synthetic process. These aspects make the *in situ* fabrication of biohybrid nanostructures poorly versatile, despite its straightforward nature, highlighting the need for alternative *ex situ* approaches.

A detailed understanding of the NP-bacterial cell interaction is essential for the rational design of efficient biohybrid systems.^23^ When presynthesized NPs are incubated with bacterial cells, the photo(electro)catalytic performance will depend on NPs size, surface chemistry, and surface charge density. Therefore, a systematic investigation of the properties of *ex situ*-synthesized NPs is crucial to elucidate the NPs-living cells interaction process.

Herein, we investigate the interaction between water-dispersible, rationally designed Au NPs with specific surface properties and metabolically active photosynthetic purple bacteria cells (*Rhodobacter capsulatus* DSMZ 152,*R. capsulatus*). We specifically examined how the surface functionalization of the NPs influences the EET process at the biotic–abiotic interface, aiming to develop biohybrid photoanodes with enhanced current generation. The NPs are engineered to be small and possess customized surface properties that allow for effective interaction with bacteria^24^ while minimizing cytotoxicity,^25−27^ thereby improving photoelectrochemical conversion and transport efficiency of photogenerated charges at the bacteria/NP/electrode interface.^28^ In this study, we strategically selected four distinct types of surface functionalization for Au NPs. The NPs were then prepared with the following functionalizations: (i) the reducing agent tetrakis(hydroxymethyl)phosphonium chloride (THPC), (ii) THPC combined with cysteine (Cys), (iii) mercaptopropionic acid (MPA), and (iv) cysteamine (CysAm). The purple bacterium *R. capsulatus* was selected due to its metabolic versatility, its potential application for H_2_ production,^29,30^ and its capability to biosynthesize and accumulate high molecular weight polymeric material (polyhydroxyalkanoates).^31^

Spectroscopic, ultrastructural (morphological), and electrochemical characterizations revealed that functionalization of Au NPs with Cys in the zwitterionic form allows uniform distribution of the NPs within the outer membrane/periplasmic space of purple bacterial cells while enhancing EET and (photo)current generation.

## Results and Discussion

### Synthesis and Characterization of Au Nanoparticles Functionalized with Small Ligands

Modification of bacterial cells with *ex situ*-synthesized NPs requires water-soluble surface functionalized and small (diameter of a few nanometers) NPs to enhance interaction with bacterial cells, while minimizing cytotoxicity or cellular stress. A small NP is compatible with the size of bacterial cellular systems, and provides a platform for finely tuning nanomaterial–bacterium interactions through appropriate surface functionalization^32^ or random internalization into the cells by either endocytosis or direct translocation.^1^ Moreover, since the portion of surface atoms increases relative to bulk atoms, interfacial processes are expected to enhance significantly. The exposed moieties of the organic shell protecting the colloidal NPs can engage in specific interactions or hydrogen bonding with membrane proteins, potentially resulting in efficient charge hopping if the shell is sufficiently thin.

The method reported by Turkevitch et al. is a popular approach to synthesize colloidally stable, citrate-capped Au NPs in an aqueous medium,^33^ offering a tunable surface chemistry. Its limitation in providing stable colloidal NPs of a few nanometers has been recently overcome^24^ by a kinetically controlled seeded-growth strategy, making use of traces of tannic acid and an excess of sodium citrate. However, high temperature conditions are still required to generate the mild reducing agents *in situ*.

In this study, NPs smaller than 5 nm were obtained via an alternative method, performed in water at room temperature following the procedure by Duff et al.^34^ which employs THPC as a mild reducing agent. Unlike the traditional approach, here, Cys is used as a multifunctional short-chain ligand, selected given its high water-solubility, environmental friendliness, affordability, and strong binding capabilities to metal surfaces. Cys is expected to preferentially bind the surface of Au NPs via the thiol group, forming S–Au bonds, while exposing the carboxylic and amine functionalities,^35^ in the zwitterionic form at physiological pH. Previous studies^36−40^ pointed out the long-term colloidal stability and low-fouling properties of zwitterionic NPs in biological applications. The addition of Cys may prevent the tendency of the NPs to coalesce,^41^ which is detrimental for processes occurring at the interface, while the thin organic shell is not expected to limit charge hopping at the NP interface.

**Figure 1:**
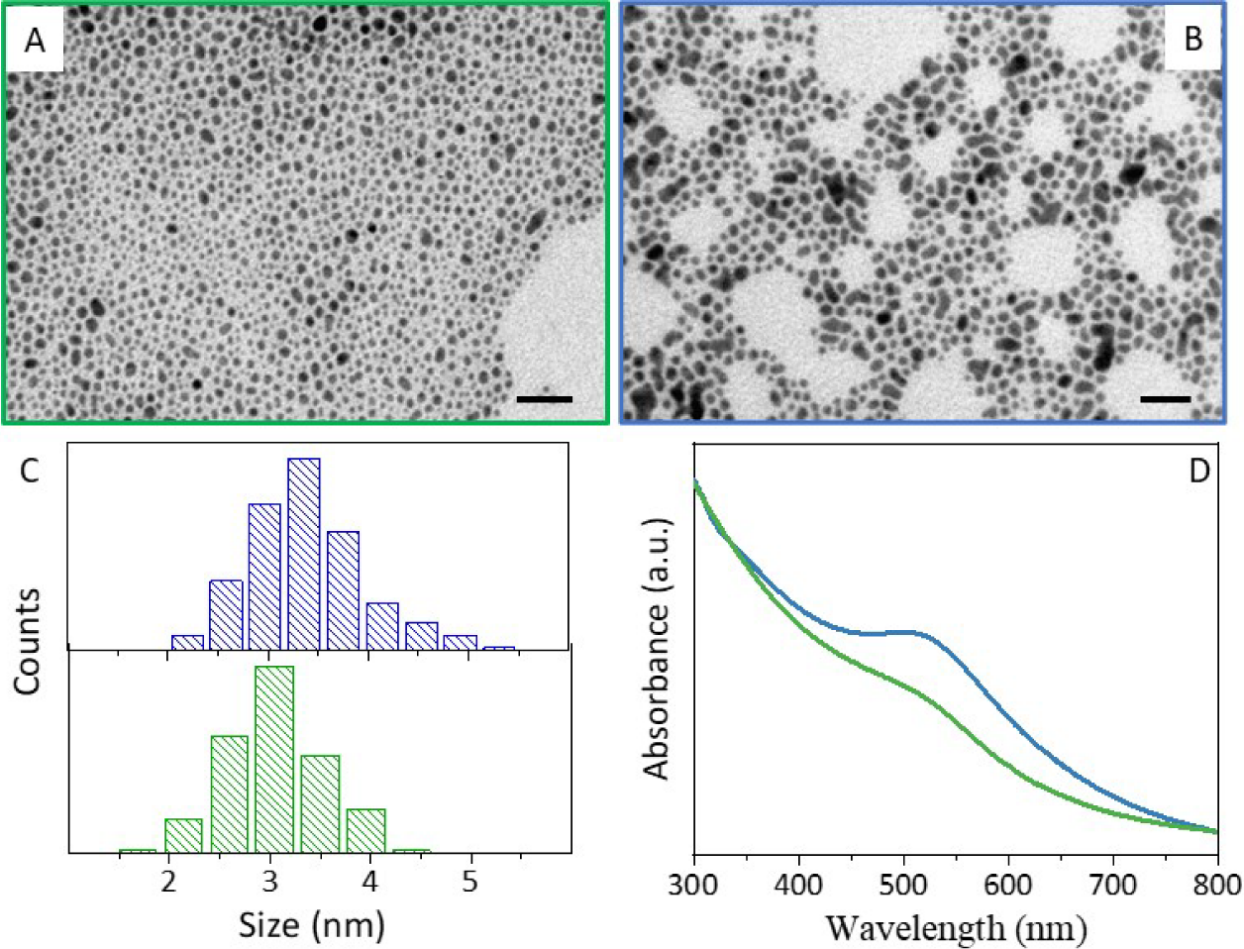
(A-B) TEM micrograph (scale bar 20 nm), (C) particle size distribution determined through statistical analysis of over 150 particles observed in TEM micrographs, and (D) UV–vis absorption spectra of Au@Cys (A, C, D color code green), Au@THPO (B, C, D color code blue) NPs.

The TEM micrograph of the as-synthesized Au@Cys NPs (Figure 1A) shows almost spherical NPs with an average size of 3.0 nm (σ% = 16%, Figure 1C, green bars). The UV–vis absorption spectrum (Figure 1D, green trace) displays the typical absorption profile for small Au NPs,^35,42,43^ with a barely visible localized surface plasmon resonance (LSPR) band at around 500 nm. Au NPs synthesized without Cys (Au@THPO NPs) were prepared as a control, showing slightly bigger NPs (Figure 1B) for an average size of 3.4 nm (σ% = 18%, Figure 1C, blue bars), featuring a more pronounced LSPR band (Figure 1D, blue trace versus green trace) than Au@Cys NPs. Furthermore, dynamic light scattering measurements were performed to estimate the hydrodynamic diameter of the particle’s dispersion (Figure S1), underlining a good agreement to the TEM values. These findings indicate that Cys used at a low Cys to THPC ratio influences NP growth.

**Figure 2:**
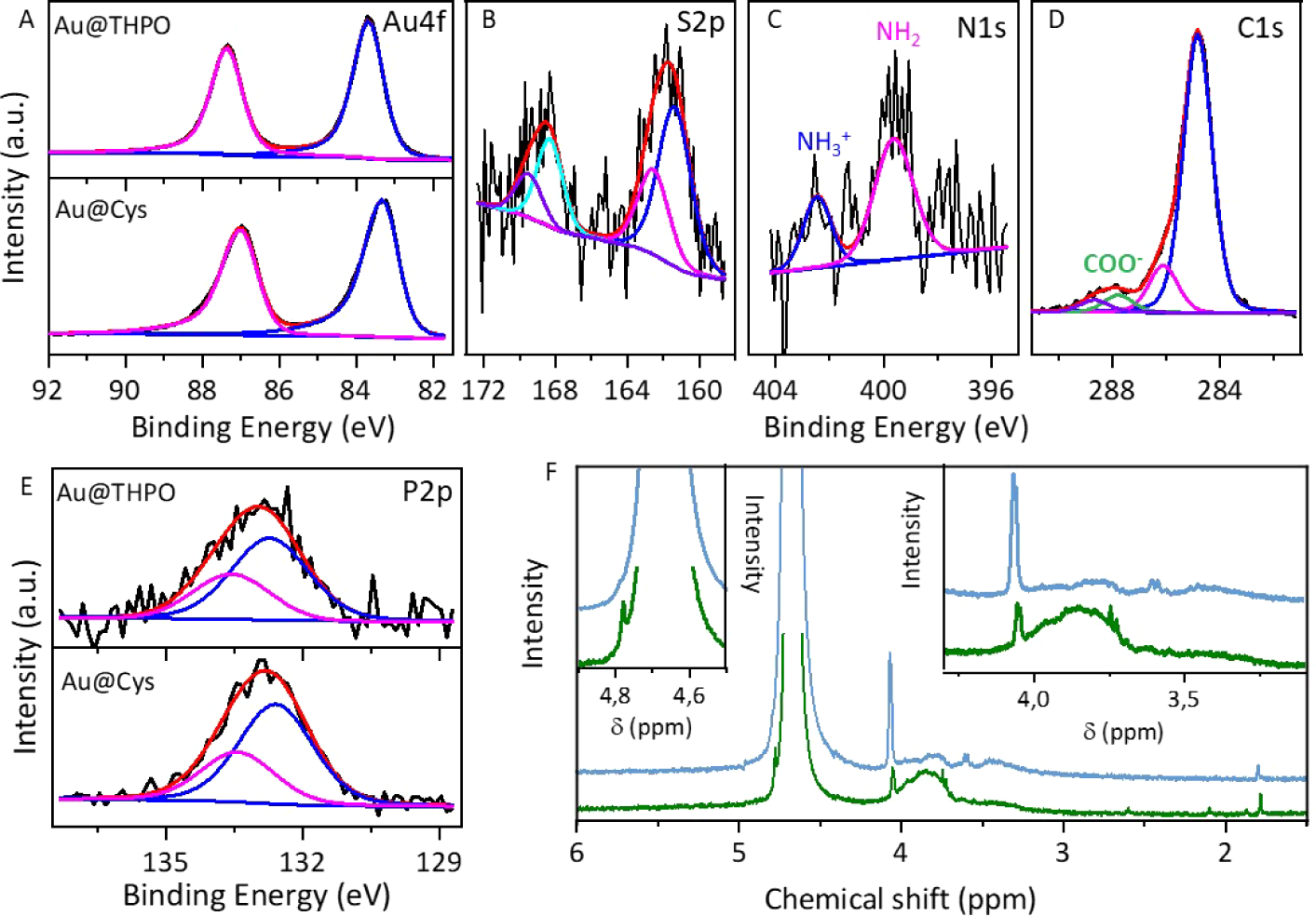
XPS high resolution spectra of Au 4*f* (A), of Au NPs samples as indicated in the figure, S 2*p* (B), N 1*s* (C), and C 1*s* (D) of Au@Cys, and P 2*p* (E) of Au NPs as indicated in the figure. ^1^H-NMR spectra (F) of Au@Cys (green line) and Au@THPO (blue line).

According to the procedure reported by Hueso et al.,^41,44,45^ in the alkaline condition of the reaction medium, THPC generates active species (formaldehyde and molecular hydrogen), which can burst the reduction of Au precursor (AuCl_4_^–^), characterized by a high reduction potential value (*E*^0^ AuCl_4_^–^/Au = 1.498 V). This process leads to the formation of nascent Au nuclei, which grow via further monomer deposition, controlled by the oxidized form of THPC, tetrakis(hydroxymethyl)phosphonium oxide (THPO). THPO acts as a stabilizing agent, binding to the metal NPs surface via a dipolar phosphorus–oxygen bond,^44^ while residual chloride and positively charged phosphonium centers contribute to the electrostatic stabilization of Au NPs. The addition of Cys to the reaction medium alters the reaction path and binding motif. The formation of Au–thiolate complexes characterized by a lower redox potential −0.25 V (versus SHE) and the strong binding affinity of the Cys thiol moiety to the preformed Au NP nuclei slow down the growth kinetic (Figure S2), resulting in NPs with a poorly pronounced LSPR band as observed for Au@Cys NPs. To pinpoint the binding functionalities and surface chemistry, X-ray photoelectron spectroscopy (XPS) and H^1^-Nuclear Magnetic Resonance (NMR) characterizations of the Au@Cys and Au@THPO samples were performed. Au 4*f* high resolution spectrum (Figure 2A) is a doublet with the main component (Au 4*f*_7/2_) peaked at 84.0 ± 0.3 eV and 83.3 ± 0.1 eV for Au@THPO and Au@Cys NPs, respectively. These binding energy values are consistent with the Au(0) oxidation state. The slight shift in binding energy between the two samples may be due to differences in the particle size as well as to the nature of the coordinating agents. The S 2*p* signal in Au@Cys NPs (Figure 2B) consists of two doublets. The one at 161.6 ± 0.1 eV (S 2*p*_3/2_), is at a lower binding energy than free thiol (S 2*p*_3/2_ at 164.2 eV) and it is indicative of the Au–S bond formation, consistently with literature.^46^ The doublet at higher binding energy (S 2*p*_3/2_ 168.4 ± 0.1 eV) is ascribed to oxidized sulfur. This component is absent in pure Cys powder (Figure S3), suggesting that Cys also acts as a reducing agent of Au ions and is oxidized *in situ* to cysteamic acid. Further insights can be gained by the analysis of the N 1*s* spectrum (Figure 2C), which displays two components: the peak at 399.6 eV, which is characteristic of free amine groups, and another one at 402.1 eV associated with NH_3_^+^. This aligns with the corresponding C 1*s* spectrum (Figure 2D), which shows both the COO^–^ and COOH functionalities, confirming the presence of Cys also in its zwitterionic form. The absence of a peak at ∼397 eV, which would indicate Au–N bonding, confirms that Cys binds to the Au NPs surface via thiol groups, with amine and carboxylic functionalities pointing outward, even in the zwitterionic form. The P 2*p* XPS signal in Au@THPO NPs (Figure 2E) consists of a doublet positioned at 133.3 ± 0.3 eV assigned to THPO. In the Au@Cys sample, the doublet downshifts to 132.7 ± 0.1 eV likely due to residual THPC. XPS quantitative analysis reveals a high chloride content in the Au@Cys NP samples compared to Au@THPO, which can be attributed to residual THPC and chlorine excess arising from the Au precursor electrostatically interacting with the ammonium functionalities of zwitterionic Cys at the Au@Cys NPs surface.

H^1^-NMR characterization (Figure 2F) supports the findings from XPS characterization. The two main signals (doublets) located at 4.16 ppm and at 3.67 ppm in the Au@THPO spectrum can be ascribed to THPO and THPOH, respectively,^41^ hinting at the complete conversion of THPC. Concerning the H^1^-NMR spectrum of Au@Cys NPs, signals at 4.7 ppm (attributable to THPC), 4.16 ppm (attributable to THPO), and a broad band in the range of 4.00÷3.50 ppm (attributable to the bound Cys) can be observed, along with the disappearance of the characteristic peaks of free Cys (Figure S3). These signals confirm that Cys binds the NP and that residual THPC along with THPO are present due to the slowdown of the overall reduction process. Additionally, characterization by Fourier transform infrared (FT-IR) spectroscopy and thermogravimetric analysis confirmed the bonding of Cys to Au NPs, and allowed us to estimate that almost 40% of the organic shell is composed of Cys, with the remaining 60% consisting of THPC and THPO functionalities (Figure S1).

**Scheme 1:**
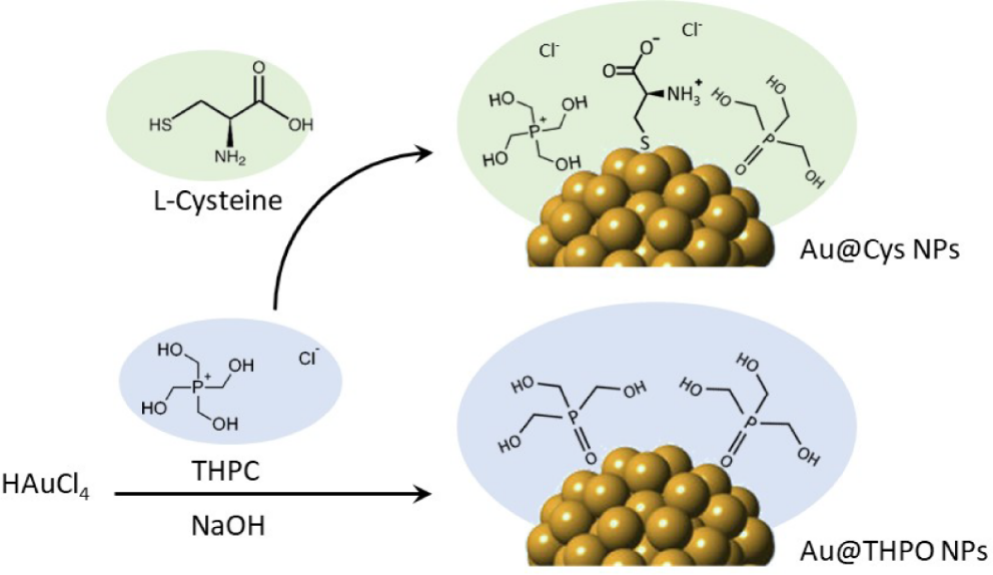
Representative Illustration of Au@THPO’s and Au@Cys NPs’ Synthetic Path and Final Surface Chemistry

Based on the comprehensive characterization, the proposed surface chemistry of Au@THPO and Au@Cys NPs is illustrated in Scheme 1. ζ potential values measured for both Au@THPO and Au@Cys NPs are −34.1 ± 1.5 and −38.7 ± 1.2 mV, respectively, indicating that both THPO and THPC are deprotonated at physiological pH.

### Incubation of *R. capsulatus* with AuNps: Vitality Test and Characterization

The modification of photosynthetic *R. capsulatus* bacteria with Au@Cys, and similarly, Au@THPO NPs, was achieved by incubating bacterial cells with NPs. Furthermore, Au NPs, synthesized by replacing Cys with MPA (Au@MPA NPs, Figures S1 and S4) or CysAm (Au@CysAm NPs, Figures S1 and S5), were also incubated with *R. capsulatus* cells. Like Cys, the addition of MPA (or CysAm) slows the kinetics of NPs growth, resulting in 3 nm-sized NPs (Figures S1 and S4). XPS characterization confirmed that MPA (or CysAm) binds to the Au NPs via its thiol group with the carboxylate moiety (or amine moiety, for Au@CysAm NPs, data not shown) pointed outward (Figure S4). The ζ potential value of −37.8 ± 0.7 mV for Au@MPA confirms the negatively charged surface of Au NPs while a ζ potential value of −28.8 ± 0.5 mV, slightly more positive than all the other samples, was measured for Au@CysAm NPs, attributable to the partial neutralization of the negative charge of the deprotonated phosphorus derivatives by the protonated amino moieties of CysAm. Therefore, incubation of Au@MPA NPs or Au@CysAm NPs with *R. capsulatus*, as control experiments, aims at clarifying the role played by surface functionalities in the interaction with bacterial cells. These controls provide insights into how carboxylic and amine groups individually drive the interaction with *R. capsulatus* and what occurs when both are present at the NP surface, as in the case of Au@Cys NPs.

**Figure 3:**
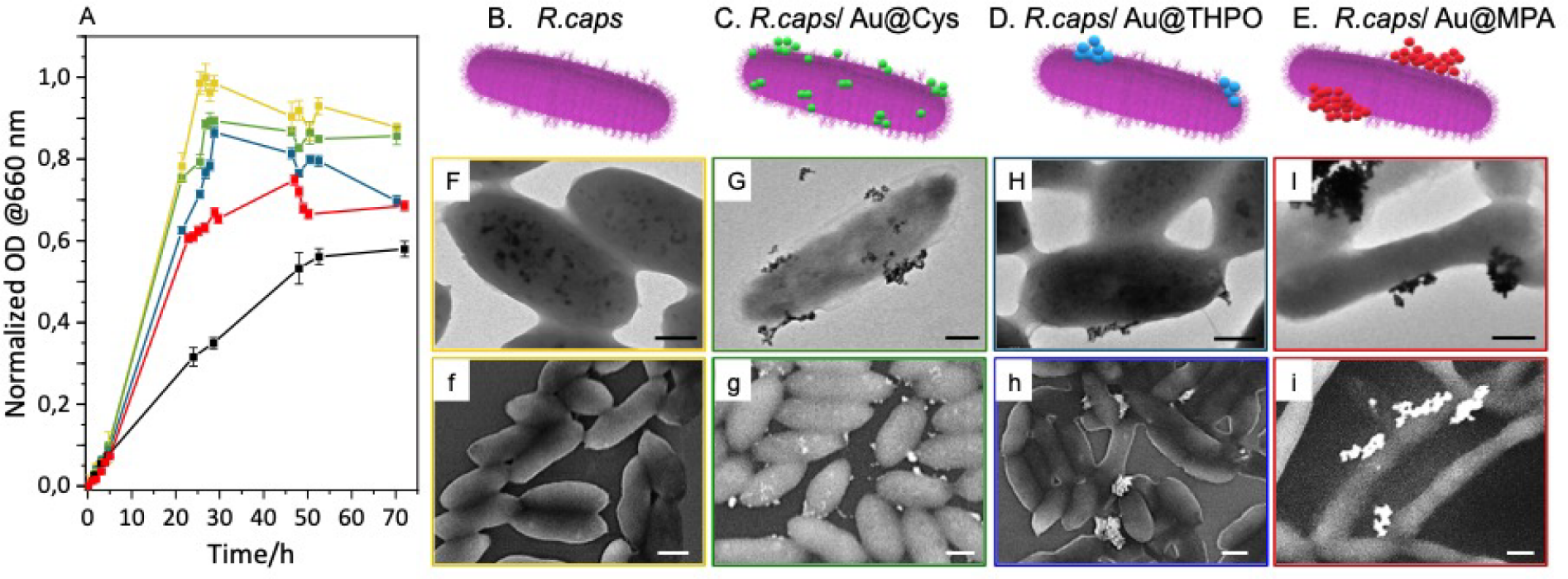
(A) Time evolution of the normalized optical density (OD) at 660 nm over 70 h for bacteria culture of *R. capsulatus* without and with Au NPs for *R. capsulatus* (yellow), *R. capsulatus*/Au@Cys NPs (green), *R.capsulatus*/Au@THPO NPs (blue), *R. capsulatus*/Au@MPA NPs (red), and *R. capsulatus*/Au@CysAm NPs (black). (B–E) Sketches, (F–I) TEM micrographs (scale bar 200 nm for F−H, 500 nm for I), and (f−i) SEM micrographs (scale bar 500 nm) of *R. capsulatus* before (B,F,f) and after incubation with Au@Cys (C,G,g), Au@THPO (D,H,h), and Au@MPA (E,I,i) NPs.

Preliminary experiments were carried out by incubating *R. capsulatus* with different NPs concentrations (Figure S6) in the growth medium to unveil potential concentration-dependent cytotoxic effects.^47,48^Figure 3A shows the bacterial vitality tests, carried out by monitoring optical density (OD) of the bacteria culture at 660 nm over 70 h for bare *R. capsulatus* and *R. capsulatus* incubated with 50 μg·mL^–1^ of Au@Cys, Au@THPO, Au@MPA, and Au@CysAm NPs. The 660 nm wavelength is conventionally used to follow bacterial growth. It falls within the spectral region of NPs transparency (Figure S7A-C), allowing the direct correlation of the recorded OD with bacterial cell density.^49^ The growth curves highlight that *R. capsulatus* incubated with the 50 μg·mL^–1^ Au@Cys NPs (*R. capsulatus*/Au@Cys NPs, Figure 3A green line) and the control sample (*R. capsulatus*, Figure 3A yellow line) reached the same OD value after 70 h, corresponding to a stationary phase where the bacterial population remains constant.

No significant differences could be observed for the exponential growth phases of *R. capsulatus* and *R. capsulatus*/Au@Cys. This result suggests that the incubation of *R.capsulatus* with these NPs does not negatively affect the vitality and replication kinetics of this bacterial strain. Conversely, prolonged latency phase and/or slower growth during the exponential phase was seen in *R. capsultatus* incubated with Au@THPO NPs (Figure 3A, blue line), Au@MPA NPs (Figure 3A red line), and Au@CysAm NPs (Figure 3A black line). Specifically, *R. capsulatus*/Au@CysAm NPs and *R. capsulatus*/Au@MPA NPs reached the two lowest OD at 30, 50, and 70 h compared to all the other cases. These results highlight the inhibitory effect, and the need for bacteria adaptation to the new environmental stresses in the sequence Au@CysAm >Au@MPA > Au@THPO NPs with respect to Au@Cys NPs.

**Figure 4:**
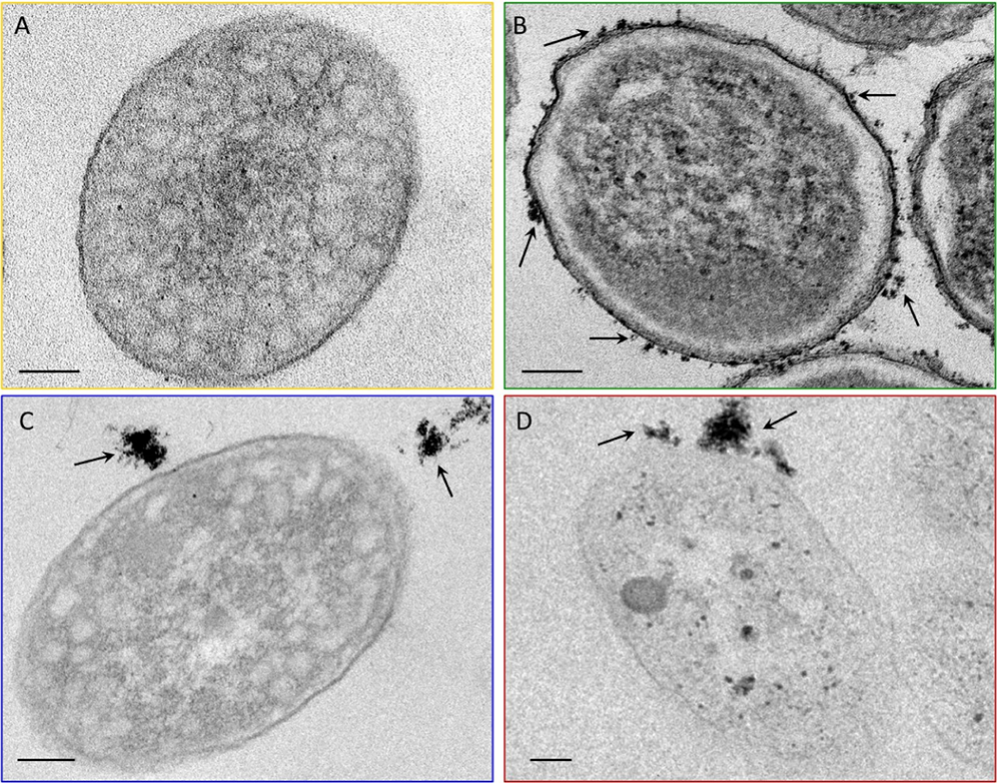
(A–D) TEM images of thin section (scale bar 100 nm) of *R. capsulatus* before (A) and after incubation with Au@Cys (B), Au@THPO (C), and Au@MPA (D) NPs.

Electron microscopy characterization by either TEM (Figures 3F–I, 4 and S5C) and SEM analysis (Figures 3f–i and S5D) of *R. capsulatus* before and after incubation with each Au NPs samples were performed to (i) monitor bacterial cells morphology, (ii) identify if cytotoxic conditions or states of bacterial distress upon exposure to Au NPs samples, and (iii) examine how Au NPs assemble on, or interact with, bacterial cells.

The wild-type *R. capsulatus* bacteria (Figure 3B,F,f) and the *R. capsulatus*/Au@Cys NPs (Figure 3C,G,g) biohybrid nanostructures, as well as *R. capsulatus*/Au@THPO (Figure 3D,H,h), show no statistically significant differences in terms of length (nearly 1.3 μm), width (nearly 0.52 μm), and aspect ratio (Figure S7D). Conversely, bacteria exposed to Au@MPA NPs (Figure 3E,I,i) and Au@CysAm NPs (Figure S5C,D) show an increased length, reaching 1.8 μm, while preserving the initial width, thus increasing the aspect ratio (Figure S7D). The elongation of purple photosynthetic bacterial cells due to exposure to environmental stress has been reported in the literature, with a similar phenomenon observed when bacterial cells are exposed to various metal ions.^50^ These findings agree with the normalized OD obtained from the growth curves, where *R. capsulatus*/Au@MPA NPs show the longest latency phase (as well as *R. capsulatus*/Au@CysAm NPs) and the lowest OD value at the stationary phase, suggesting a more pronounced cytotoxic effect. The different contrast between dehydrated bacteria and metal NPs helps unveiling the localization of the NPs and, thanks to energy dispersive X-ray spectroscopy (Figure S8) analysis, the chemical composition of the observed morphological features can be determined. Regardless of the Au NPs samples, the TEM micrographs reported in Figure 3G–I show dark spots attributed to Au NPs generally assembled into aggregated structures decorating the bacterial cells. SEM images of the same samples, recorded over a large sample area (Figure 3g–i) show Au@Cys NPs (Figure 3g) widely distributed and characterized by a lower degree of aggregation compared to Au@THPO NPs (Figure 3h) and Au@MPA NPs (Figure 3i) samples. TEM images of the thin sections of the bacterial cells (Figure 4) highlight a very dark outline of *R. capsulatus* cells and NPs located in the periplasmatic space along with small aggregates, densely distributed on the cell surface for Au@Cys NPs (Figure 4B). Conversely, Au@THPO NPs (Figure 4C) appear aggregated in bigger nanostructures rather distant from the bacterial cell membrane and embedded in the exopolysaccharide matrix, resulting in a poor interaction with *R. capsulatus* cells. For Au@MPA NPs, aggregates decorating the bacterial cells membrane are obtained. It can be thus concluded that the zwitterionic surface of Au@Cys NPs ensures longer colloidal stability during the bacterial cells growth stage, which may be beneficial for enhancing interaction and promoting widespread-distributed NPs assembly and NP internalization within the bacterial cells membrane, meanwhile not resulting cytotoxic. Conversely, large aggregates adhering to the bacterial membrane as in the case of *R. capsulatus*/Au@MPA NPs biohybrid cause a stressed condition limiting cell vitality. Based on the obtained TEM and SEM analyses it is possible to determine the average amount of Au@Cys NPs per bacterial cells in 600 ± 250. Such a determination is not performed for NPs with different functionalization since the presence of large aggregates would make the quantification highly variable.

### Electrochemical Test for *R. capsulatus* Biophotoanodes

**Figure 5:**
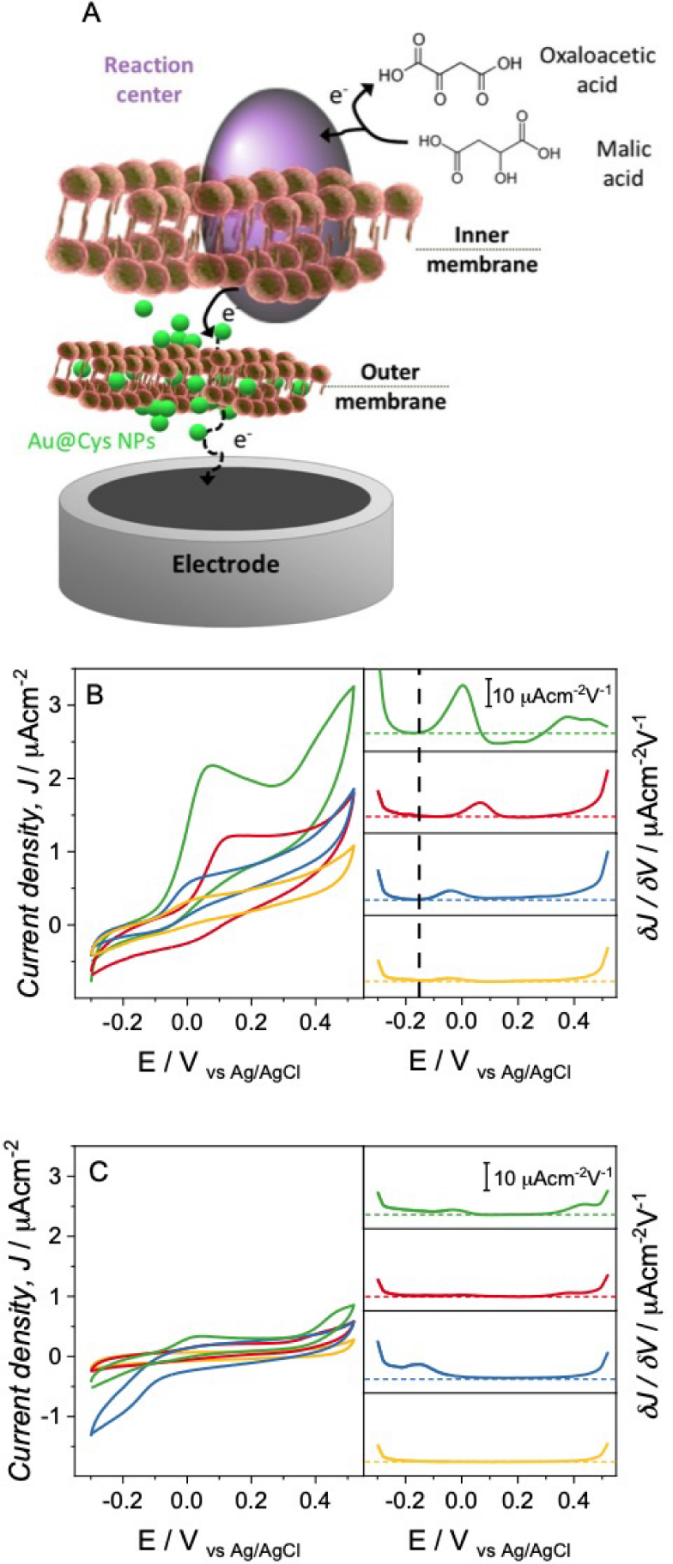
(A) Scheme of the extracellular electron transfer pathways enhanced by Au NPs adapted from ref.^54^ with permission from the European Society for Photobiology, the European Photochemistry Association, and the Royal Society of Chemistry. (B–C) Left panels: Cyclic voltammetries of *R. capsulatus* (yellow line), and *R. capsuslatus* incubated with Au@Cys NPs (green line), Au@THPO NPs (blue line), and Au@MPA NPs (red line) under illumination (B) and in dark conditions (C) at a scan rate of 2 mV·s^–1^. Right Panels: First derivative of the cyclic voltammetries.

The performance of Au NPs-modified bacterial biohybrid systems in converting visible light into electricity was evaluated by electrochemical characterization carried out in a standard three-electrode system equipped with a fiber optic light. Figure 5A illustrates the biohybrid system and the light-activated mechanisms triggered by the photosynthetic bacteria, highlighting the expected role of the NPs in the electron transfer process at the biotic/abiotic interface. Electrochemical tests were performed to decipher whether the different assembly of NPs on the bacterial cell influences electrochemical performance. The thin organic shell passivating the Au NPs surface is expected not to detrimentally impact the charge transport at the bacterial cell-NP interface.

Figure 5B shows the cyclic voltammetries (CVs, left panel) and their first derivative (right panel) performed under illumination for all of the prepared biophotoelectrodes. Interestingly, considerably different behavior was obtained for *R. capsulatus*/Au@Cys NPs, *R. capsulatus*/Au@MPA NPs, and *R. capsulatus*/Au@THPO NPs. First, the electrode prepared with *R. capsulatus*/Au@Cys NPs (Figure 5B green line) showed the lowest onset potential for the anodic reaction (−0.16 ± 0.02 V), as highlighted by the dashed black line in the first derivative of the cyclic voltammetry, and achieved a current density of 1.5 ± 0.2 μA·cm^–2^ at +0.2 V. Conversely, *R. capsulatus*/Au@MPA NPs (Figure 5B red line) exhibited an onset potential shifted to considerably more positive values (+0.03 ± 0.01 V) indicating a less favorable EET process, together with lower current densities at +0.2 V (0.8 ± 0.2 μA cm^–2^). It is important to consider that the lower current density achieved might be due to an inhibitory effect of Au@MPA NPs on bacterial metabolism that negatively affects the oxidation of the electron donor (malic acid). *R. capsulatus*/Au@THPO NPs (Figure 5B blue line) showed an onset potential slightly higher than the one of *R. capsulatus*/Au@Cys NPs (−0.14 ± 0.01 V), but achieved considerably lower current densities, suggesting that the nonuniform distribution of NPs does not efficiently facilitate EET. The electrodes prepared with wild-type bacterial cells (Figure 5B, yellow line) achieved both a lower current density and a higher onset potential, in agreement with previous studies reporting limited EET capability for these bacteria.^51,52^ The biotic origin of the obtained current was confirmed by performing CVs using heat-treated bacteria at 80 °C (Figure S9), which resulted in the absence of significant current generation, thus confirming the role of metabolically active bacteria in the biophotoelectrode performance. The CVs performed in dark conditions and their first derivatives (Figure 5C left and right, respectively) remark the same trend observed for the CVs performed under illumination but with lower current densities for all of the electrodes. The possibility to obtain current generation also in the absence of illumination has been previously reported for *R. capsulatus* electrodes,^52,53^ and stems from the conventional respiration mechanisms of these bacteria in the absence of light (heterotrophic metabolism).

**Figure 6:**
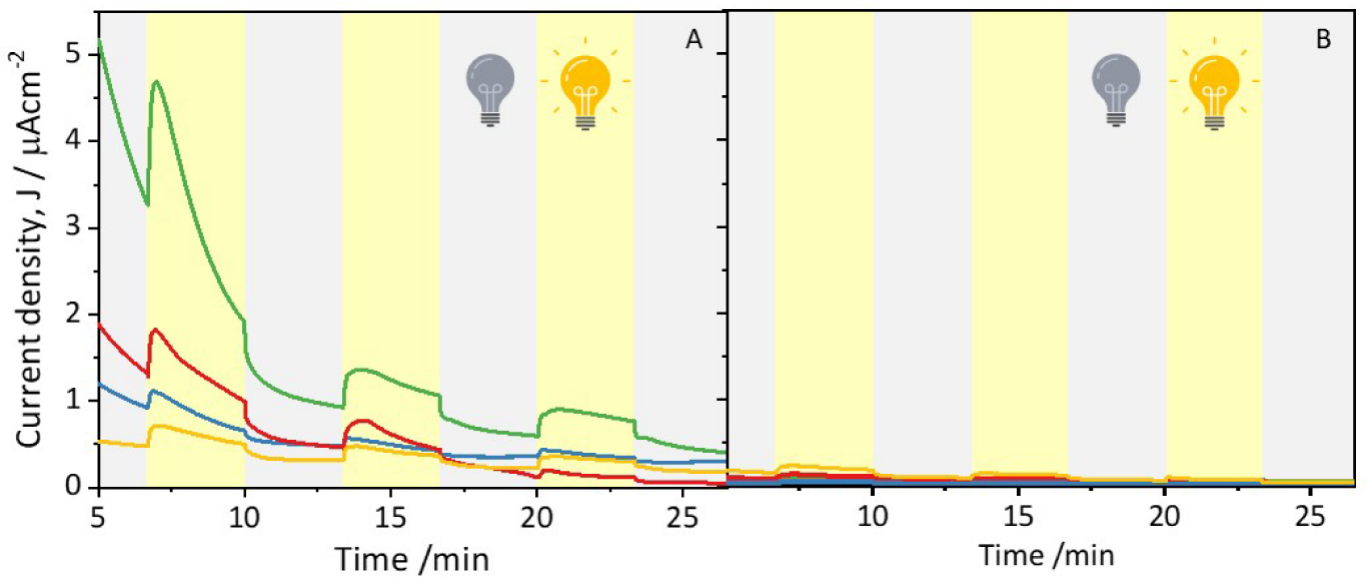
Amperometric *I*–*t* curves at +0.32 V versus Ag|AgCl 3 M NaCl under light/dark conditions for (A) *R. capsulatus* (yellow trace) and R. capsulatus incubated with Au@Cys NPs (green trace), Au@THPO NPs (blue trace), and Au@MPA NPs (red trace), and (B) thermally treated samples.

To further investigate the impact of bacterial modification with each Au NPs sample on the photoelectrochemical performance, the photocurrent generation over time was studied by chronoamperometry (Figure 6). A potential of +0.32 V was selected based on the performed CVs to ensure a sufficient overpotential to drive the anodic reaction for the various photoelectrodes prepared with the bacteria with different NPs. Immediately after the start of the polarization, during the first dark cycle, all the electrodes showed an initial drop in current density, due to the decrease of the capacitive contribution to the current response.

For a proper comparison of the different electrodes, the third illumination cycle was considered, where a *quasi*-steady-state current was obtained. As previously seen for the CV studies, also the amperometric *I*–*t* curves confirmed that *R. capsulatus*/Au@Cys NPs achieved the highest current generation (0.70 ± 0.09 μA·cm^–2^) compared to all the other electrodes, with a photocurrent of about 0.4 μA·cm^–2^ (light minus dark current). Interestingly, *R. capsulatus*/Au@MPA NPs showed a sharp decrease in the current generation, approaching zero after 25 min, further indicating the potential inhibitory effect of MPA functionalization on bacterial metabolism. It should be noted that for this chronoamperometry study a relatively high potential was utilized (+0.32 V) to ensure that a sufficient overpotential for the anodic reaction was provided for all the different microbial photoelectrodes. However, such overpotential might induce an oxidative stress on the bacterial cells, which could be one of the causes of the observed current decrease over time. While this work was not aimed at optimizing the long-term stability of the microbial photoelectrodes, future studies should be directed toward defining the optimal operating conditions to improve this aspect. Additionally, comparing the current densities achieved in the present study for the best performing photoelectrode configuration (purple bacteria modified with Au@Cys NPs) with those from literature studies using various photosynthetic bacteria (purple bacteria and cyanobacteria) shows that our values fall within the same range (a few μA·cm^–2^). However, it is important to remark that (i) the present study did not use a diffusible redox mediator, which simplifies the implementation of the biohybrid system in real-world environments by avoiding the release of mediator into the environment,^49,51,53^ and (ii) a relatively flat electrode (glassy carbon) was used. Studies employing more porous electrodes (i.e., graphite or macroporous electrodes)^52^ might achieve higher current density when the projected geometrical area is considered for its calculation, as underlined in the work by Wenzel et al.^55^ Furthermore, the biohybrid electrodes prepared with *R. capsulatus* modified with Au@cys NPs exhibited a significantly lower onset potential for the anodic photocurrent (−0.16 ± 0.02 V vs Ag|AgCl) compared to systems using purple bacteria with redox mediators reported in literature (redox potentials ranging from +0.1 to +0.25 V vs Ag|AgCl).^49,51−53^ Given these points, the procedure that we reported, which directly enhances the EET process to facilitate electron transfer from the bacteria to the electrode, represents a critical improvement that could potentially be applied to different electrode configurations to maximize current densities.

## Conclusions

Various studies have reported the use of inorganic NPs interfaced with bacterial cells to obtain biohybrid electrodes with enhanced electrochemical performance; however, detailed insights into NP–cell interaction remain limited. Here, we have synthesized *ex situ* water dispersible Au NPs of nearly 3 nm, functionalized with different short ligands, and investigated the light-to-electricity conversion of *R. capsulatus*/Au NPs biohybrids as photoanodes. By determining the NPs surface charge and chemistry by complementary spectroscopic techniques, together with morphological and electrochemical characterizations, we deciphered the role of surface functionalities in affecting bacteria modification with NPs and their photoelectrochemical performances. It was observed that, when biohybrids are formed by large NP aggregates adhering to bacterial cells, an inhibitory effect in microorganism growth is obtained concomitantly to poor electrochemical response. Conversely, no adverse effects on bacterial vitality were obtained when smaller aggregates decorate the bacterial cell surface and NPs are partially located in the periplasmatic space as a consequence of colloidal stability of NPs (as for *R. capsulatus*/Au@Cys NPs biohybrid), resulting in the highest photocurrent generation together with the lowest anodic onset potential, both indicating efficient EET processes.

The rational deciphering of the role played by the NP surface chemistry in enhancing the bacterial cells-NP interactions by promoting the homogeneous distribution of Au NPs both outside and within the membrane, and avoiding cytotoxic conditions, has effectively improved the electrochemical performance in biohybrid systems. While the biohybrid system developed in this work has been tested as a biophotoanode for current generation, the enhanced EET process achieved by Au@Cys NPs is of critical relevance also for the development of biophotocathodes, broadening the application of the systems to H_2_ and polyhydroxyalkanoates production. Accordingly, this work paves the way toward understanding these mechanisms and future studies aimed at developing novel strategies to harness microbial activities for renewable energy production and environmental remediation.

## Materials and Methods

### Materials

All chemicals were used as received without further purification. Milli-Q grade water was used for the preparation of all aqueous solutions. Tetrahydrate tetrachloroauric acid (HAuCl_4_·3H_2_O), tetrakis(hydroxymethyl)phosphonium chloride (THPC, 80% in water), sodium hydroxide (NaOH), l-cysteine (Cys), mercaptopropionic acid (MPA), ethanol (99.8%), glutaraldehyde solution (25% in water), ethylenediaminetetraacetic acid (EDTA), ammonium sulfate ((NH_4_)_2_SO_4_), magnesium sulfate heptahydrate (MgSO_4_·7H_2_O), malonic acid, calcium chloride dihydrate (CaCl_2_·2H_2_O), ferrous sulfate heptahydrate (FeSO_4_·7H_2_O), thiamine, biotin, dipotassium hydrogen phosphate (K_2_HPO_4_), potassium dihydrogen phosphate (KH_2_PO_4_), boric acid (H_3_BO_3_), manganese(II) sulfate (MnSO_4_), sodium molybdate dihydrate (Na_2_MoO_4_·2H_2_O), zinc sulfate heptahydrate (ZnSO_4_·7H_2_O), copper(II) nitrate trihydrate (Cu(NO_3_)_2_·3H_2_O), cobalt(II) chloride (CoCl_2_), 3-(*N*-morpholino)propanesulfonic acid (MOPS), magnesium chloride hexahydrate (MgCl_2_·6H_2_O), osmium tetroxide 4% solution, sodium cacodylate trihydrate, propylene oxide, Phosphate buffered saline (PBS), Embed 812 kit, UranyLess EM stain, Reynolds lead citrate 3% EM stain, and glutaraldehyde were used as chemical reagents. The bacteria used in all experiments belong to the order Rhodobacterales, family Rhodobacteraceae, species *Rhodobacter capsulatus* DSMZ 152, obtained from Deutsche Sammlung von Mikroorganismen and Zellkulturen GmbH (DSMZ).

## Methods

### Synthesis of Functionalized Au Nanoparticles

The synthesis of Au@THPO NPs was performed following Pham et al.^45^ procedure with minor modifications. In a 100 mL round-bottom flask were added 40 mL of milli-Q water, 0.5 mL of 1 M NaOH, and 1 mL of THPC solution, prepared by diluting 12 μL of THPC solution in 1 mL of milli-Q water (67 mmol of THPC). The reaction mixture was stirred for 10 min at room temperature before adding 5.0 mL of HAuCl_4_ 10 mM. For the synthesis of Au@Cys NPs, Au@MPA NPs and Au@CysAm NPs, the same procedure was followed. However, right after the addition of the gold precursor solution, 0.5 mL of a 10 mM aqueous solution at pH 11 of Cys or MPA or CysAm (5 μmol) was added, respectively. The prepared solutions were left stirring overnight at room temperature and then ultracentrifuged at 100,000 × *g* for 4 h at 4 °C. The collected pellet was redispersed in 1 mL of Milli-Q water and stored in the refrigerator at 4 °C for further use. The concentration of NPs was determined by freeze-drying aliquot of the sample, resulting in approximately 8 mg·mL^–1^.

### Bacterial Cell Viability Tests

*R. capsulatus* cells were grown in sterile 8 mL vials, sealed with airtight caps, in the presence of a liquid culture medium. One L of liquid culture medium was prepared by adding 20 mg of EDTA, 1.0 g of (NH_4_)_2_SO_4_, 4.0 g of malic acid, 200 mg of MgSO_4_·7H_2_O, 75 mg of CaCl_2_·2H_2_O, 12 mg of FeSO_4_·7H_2_O, 1 mg of thiamine, 15 mg of biotin, 0.9 g of K_2_HPO_4_, 0.6 g of KH_2_PO_4_, 1 mL of trace element solution, and Milli-Q water to reach the desired volume. The composition of 250 mL of the trace element solution is as follows: 700 mg of H_3_BO_3_, 398 mg of MnSO_4_, 188 mg of Na_2_MoO_4_·2H_2_O, 60 mg of ZnSO_4_·7H_2_O, 10 mg of Cu(NO_3_)_2_·3H_2_O, and 50 mg of CoCl_2_, along with Milli-Q water, to reach the desired volume. The pH of the medium was adjusted to 6.8 with 5 M NaOH before sterilization at 125 °C for 25 min in an autoclave (Systec VX-55). The trace element solution, MgSO_4_, CaCl_2_, FeSO_4_, and biotin are added to the culture medium after sterilization by filtration using a 0.20 μm filter (Puradisc 25) in a sterile hood.

For the viability test, bacterial cells were exposed to light radiation produced by an 80 W incandescent lamp and maintained at a temperature of 28 °C inside an incubator (IKA KS 3000 i control). A typical bacterial growth solution consisted of a 10% stock solution of *R. capsulatus*, the required volume of NP solution to achieve a final concentration of 10, 50, or 100 μg·mL^–1^, and a liquid culture medium to reach the desired volume. The aqueous NP dispersions were sterilized by UV irradiation before being used for growth solution preparation, which was carried out in a biological hood. Bacterial growth was monitored by measuring the optical density (OD) by using a turbidimeter equipped with a 660 nm filter. For the determination of the standard growth curve of bacteria not exposed to NPs, a culture of *R. capsulatus* without the addition of NP solution was used as reference.

### Preparation of the Bioanode

*R. capsulatus* cells modified with Au NPs or wild type *R. capsulatus* cells, grown in a 50 mL batch for 72 h were collected by centrifugation at 4000 × g for 20 min at 20 °C (260R MPV). The resulting cell pellet was resuspended in 1 mL of electrolyte solution and centrifuged again at 9700 rpm for 10 min at room temperature (DLAB D2012plus). Subsequently, the obtained pellet was resuspended in an electrolyte solution to achieve a concentration of 1 g·mL^–1^. The utilized electrolyte was 20 mM MOPS + 10 mM MgCl_2_·6H_2_O + 50 mM malic acid (pH adjusted to 7 using 5 M NaOH). The bioanode was prepared by drop-casting 5 μL of the bacterial solution onto a glassy carbon electrode (BASI, MF-2012), and allowing the electrode to dry for 20–30 min prior to the electrochemical characterization.

### Bacterial Preparation for TEM Investigation

A solution was prepared by combining 200 μL of a bacterial culture solution, 700 μL of 0.1 M sodium cacodylate buffer, and 100 μL of a 25% aqueous glutaraldehyde solution. The resulting solution was refrigerated overnight to ensure proper fixation of the bacterial samples. Subsequently, a dehydration process was carried out to prepare the samples for the TEM investigation. Each dehydration step involved centrifuging the cell pellet at 9700 × *g* for 15 min using a DLAB D2012plus centrifuge. After centrifugation, the pellet was carefully resuspended in 200 μL of an aqueous ethanol solution with an increasing concentration. The dehydration cycles were performed by sequentially increasing the volumetric percentage of ethanol (25%, 50%, 75%, 95%, and 100%) during a 10 min incubation period for each step. After the last washing step, the ethanol solution containing the dehydrated samples was drop-cast onto a TEM grid, allowing the solvent to evaporate. At this stage, the sample is ready for initial TEM analysis. For thin section analysis, the dehydrated samples were centrifuged at 9700 × *g* for 15 min to obtain a cell pellet at the bottom of the Eppendorf tube. The pellet was then postfixed in 0.1 M sodium cacodylate buffer at pH 7.4 containing 1% osmium tetroxide and gently stirred for 2 h. The samples were then washed three times in the cacodylate buffer for 10 min, followed by two washes in Milli-Q water for 5 min. The samples underwent sequential dehydration in graded ethanol (70, 90, 96, and 100% v/v). Each step was repeated twice. After ethanol dehydration, they were washed twice with propylene oxide, embedded in epoxy resin (Epon 812, TAAB) and sliced using a diamond blade (Diatom) with an ultramicrotome (UC6, Leica). The slices were then stained with a drop of UranyLess and Reynolds lead citrate stain before TEM observation.

### Characterizations

The UV–vis absorption spectra were recorded using a Cary 5000 UV/vis/NIR spectrophotometer (Varian, Leini, TO, Italy). For acquiring the spectrum of Au NP samples, they were diluted with Milli-Q water 1:20 before purification and 1:250 after purification.

Transmission electron microscopy (TEM) characterization was performed by using a JEOL JEM 1011 transmission electron microscope operating at 100 kV and equipped with a Gatan Orius SC1000 series CCD camera (4008 × 2672 active pixels). Statistical analysis of NP sizes in the samples and their respective distributions was conducted using the image analysis software ImageJ. For each sample, the average NP size and the relative percentage standard deviation (σ%) were calculated. The dehydrated (un)modified bacteria cell samples and the thin sections were also characterized.

For dynamic light scattering (DLS) and ζ-potential measurements, the Malvern Zetasizer nano ZSP instrument was used, equipped with a 50 mW diode laser operating at a wavelength of 532 nm. For the analysis, a solution of 7 μg·mL^–1^ in filtered ultrapure water was used.

^1^H-NMR spectra were recorded on an Agilent 500 MHz DD2 NMR instrument.

Surface chemical composition was investigated by XPS analyses with a PHI 5000 Versa Probe II spectrometer (Physical Electronics) equipped with a monochromatic Al Kα X-ray source (1486.6 eV) with a beam diameter of 200 μm. Survey (0–1400 eV) and high-resolution spectra were recorded in FAT (Fixed Analyzer Transmission) mode at pass energy of 117.40 and 29.35 eV, respectively. Surface charging was compensated for using a dual beam charge neutralization system. The hydrocarbon component of C 1*s* spectrum was used as an internal standard for charging correction, and it was fixed at 284.8 eV. On each sample, the analysis was repeated on five different spots to assess treatment homogeneity. Spectra were processed with MultiPak software (Physical Electronics).

Field emission scanning electron microscopy (FE-SEM) was performed by using a Zeiss Sigma Microscope (Carl Zeiss Co., Oberkochen, Germany) operating in the range 0.5–20 keV and equipped with an in-lens and secondary electron detectors. SEM micrographs were acquired by mounting the TEM grid on the stab using carbon tape. Measurements were carried out at an operating voltage of 5 keV and a working distance of 4.5 mm. Elemental composition was obtained by energy-dispersive X-ray spectroscopy (EDS, INCA Energy by Oxford Instruments Analytical) using an accelerating voltage of 20 keV and a working distance of 7 mm.

The electrochemical characterization of the prepared biophotoelectrodes was performed by cyclic voltammetry and chronoamperometry in a classical three-electrode electrochemical cell connected to a potentiostat (PalSens4). The working electrode consisted of the different bioanodes, and the control electrodes were obtained as described earlier. The counter electrode was a platinum wire, and the reference electrode was a Ag|AgCl electrode (3 M NaCl, BASI MF-2052). All potentials reported in this work refer to this reference electrode unless differently specified. Control experiments were conducted using as control electrodes glassy carbon in the absence of *R. capsulatus* cells or with bacterial cells that were heat-treated to render them metabolically inactive. 30 mL of an electrolyte composed of 20 mM MOPS + 10 mM of MgCl_2_·6H_2_O + 50 mM malic acid (pH adjusted to 7 using 5 M NaOH) were utilized in the electrochemical cell. Malic acid is an ideal carbon source for *R. capsulatus*, and the used concentration approaches substrate saturation, as reported in literature.^49,52^ Before proceeding with the analyses, Argon gas was purged into the electrolyte solution for 20 min to obtain anaerobic conditions. Illumination during the cyclic voltammetry experiments was provided by a fiber optic lamp (Schott KL 1500 LCD) with a 10 W bulb, providing an output light intensity of 125 mW·cm^–2^. CVs were performed in a potential window of −0.3 to +0.52 V, and the scan rate was 2 mV·s^–1^. The chronoamperometries were performed at +0.32 V alternating light/dark cycles every 200 s. At least three independent replicate experiments were performed for all of the conditions investigated, and average values are reported, together with one standard deviation.

TGA was carried out using a Pyris 1-PerkinElmer instrument under a nitrogen flow of 40 mL·min^–1^ at the heating rate of 20 °C·min^–1^ in a temperature range from 75 to 700 °C.

Thermograms were collected using dried NP samples.

The FTIR characterization was carried out using a Varian 670 FTIR spectrometer equipped with a diamond ATR accessory of 2 mm and a deuterated tryglicine sulfate detector. One μL of each sample and the powder, in the case of Cys, were put on the internal reflection element, and the solvent was let to evaporate. Spectra were recorded in the range 4000–400 cm^–1^ acquiring 16 scans with a nominal resolution of 1 cm^–1^.

## Data Availability

The data supporting this article have been included in the manuscript and as part of the Supporting Information.

## Supporting Information Available

DLS, FT-IR, and thermogravimetric analyses. UV–vis absorption spectra of colloidal solutions. XPS high resolution and ^1^H NMR spectra. Spectroscopic and morphologic characterization of Au@MPA NPs. Spectroscopic and morphologic characterization of Au@CysAm NPs. Time evolution of the normalized optical density for bacterial growths. UV–vis–NIR absorption spectra and size distribution analysis of wild type and modified bacteria exposed to NPs. Energy dispersive X-ray spectroscopy of wild type and modified bacteria exposed to NPs. Cyclic voltammograms for control electrodes (PDF)

## References

[ref1] Shin Y.; Lim Y.; Lee A. R.; Lee L. P.; Kim D.; Cho M.-L.; Kang T. Electron-Transport-Chain-Mediated Selective Growth of Gold Nanocrystals in the Intermembrane Space of Live Microbial Cells. ACS Nano 2024, 18, 10045–10053. 10.1021/acsnano.3c11776. 38527965

[ref2] Elhadad A.; Liu L.; Choi S. Plug-and-play modular biobatteries with microbial consortia. J. Power Sources 2022, 535, 231487 10.1016/j.jpowsour.2022.231487.

[ref3] Noori M. T.; Thatikayala D.; Pant D.; Min B. A critical review on microbe-electrode interactions towards heavy metal ion detection using microbial fuel cell technology. Bioresour. Technol. 2022, 347, 126589 10.1016/j.biortech.2021.126589. 34929327

[ref4] Szydlowski L.; Lan T. C. T.; Shibata N.; Goryanin I. Metabolic engineering of a novel strain of electrogenic bacterium Arcobacter butzleri to create a platform for single analyte detection using a microbial fuel cell. Enzyme Microb. Technol. 2020, 139, 109564 10.1016/j.enzmictec.2020.109564. 32732044

[ref5] Adekunle A.; Gomez Vidales A.; Woodward L.; Tartakovsky B. Microbial fuel cell soft sensor for real-time toxicity detection and monitoring. Environ. Sci. Pollut. Res. 2021, 28, 12792–12802. 10.1007/s11356-020-11245-6. 33089465

[ref6] Lazzarini Behrmann I. C.; Grattieri M.; Minteer S. D.; Ramirez S. A.; Vullo D. L. Online self-powered Cr(VI) monitoring with autochthonous Pseudomonas and a bio-inspired redox polymer. Anal. Bioanal. Chem. 2020, 412, 6449–6457. 10.1007/s00216-020-02620-w. 32270248

[ref7] Dong F.; Lee Y. S.; Gaffney E. M.; Liou W.; Minteer S. D. Engineering Cyanobacterium with Transmembrane Electron Transfer Ability for Bioelectrochemical Nitrogen Fixation. ACS Catal. 2021, 11, 13169–13179. 10.1021/acscatal.1c03038.

[ref8] Zhang K.; Zhou Y.; Song T.; Xie J. Bioplastic Production from the Microbial Electrosynthesis of Acetate through CO2 Reduction. Energy Fuels 2021, 35, 15978–15986. 10.1021/acs.energyfuels.1c02594.

[ref9] Bajracharya S.; Yuliasni R.; Vanbroekhoven K.; Buisman C. J.; Strik D. P.; Pant D. Long-term operation of microbial electrosynthesis cell reducing CO(2) to multi-carbon chemicals with a mixed culture avoiding methanogenesis. Bioelectrochemistry 2017, 113, 26–34. 10.1016/j.bioelechem.2016.09.001. 27631151

[ref10] Weliwatte N. S.; Grattieri M.; Minteer S. D. Rational design of artificial redox-mediating systems toward upgrading photobioelectrocatalysis. Photochem. Photobiol. Sci. 2021, 20, 1333–1356. 10.1007/s43630-021-00099-7. 34550560 PMC8455808

[ref11] Buscemi G.; Trotta M.; Vona D.; Farinola G. M.; Milano F.; Ragni R. Supramolecular Biohybrid Construct for Photoconversion Based on a Bacterial Reaction Center Covalently Bound to Cytochrome c by an Organic Light Harvesting Bridge. Bioconjugate Chem. 2023, 34, 629–637. 10.1021/acs.bioconjchem.2c00527. PMC10120590 36896985

[ref12] Buscemi G.; Vona D.; Stufano P.; Labarile R.; Cosma P.; Agostiano A.; Trotta M.; Farinola G. M.; Grattieri M. Bio-Inspired Redox-Adhesive Polydopamine Matrix for Intact Bacteria Biohybrid Photoanodes. ACS Appl. Mater. Interfaces 2022, 14, 26631–26641. 10.1021/acsami.2c02410. 35639658 PMC9204692

[ref13] Bird L. J.; Kundu B. B.; Tschirhart T.; Corts A. D.; Su L.; Gralnick J. A.; Ajo-Franklin C. M.; Glaven S. M. Engineering Wired Life: Synthetic Biology for Electroactive Bacteria. ACS Synth. Biol. 2021, 10, 2808–2823. 10.1021/acssynbio.1c00335. 34637280

[ref14] Atkinson J. T.; Su L.; Zhang X.; Bennett G. N.; Silberg J. J.; Ajo-Franklin C. M. Real-time bioelectronic sensing of environmental contaminants. Nature 2022, 611, 548–553. 10.1038/s41586-022-05356-y. 36323787

[ref15] Cao B.; Zhao Z.; Peng L.; Shiu H.-Y.; Ding M.; Song F.; Guan X.; Lee C. K.; Huang J.; Zhu D. Silver nanoparticles boost charge-extraction efficiency in *Shewanella* microbial fuel cells. Science 2021, 373, 1336–1340. 10.1126/science.abf3427. 34529487

[ref16] Han H.-X.; Tian L.-J.; Liu D.-F.; Yu H.-Q.; Sheng G.-P.; Xiong Y. Reversing Electron Transfer Chain for Light-Driven Hydrogen Production in Biotic–Abiotic Hybrid Systems. J. Am. Chem. Soc. 2022, 144, 6434–6441. 10.1021/jacs.2c00934. 35377628

[ref17] Mouhib M.; Antonucci A.; Reggente M.; Amirjani A.; Gillen A. J.; Boghossian A. A. Enhancing bioelectricity generation in microbial fuel cells and biophotovoltaics using nanomaterials. Nano Res. 2019, 12, 2184–2199. 10.1007/s12274-019-2438-0.

[ref18] Guo X.; Yang C.; Wu J.; Ning W.; Wang T.; Wang R.; Liu S.; Li J.; Chen Z.; Li S. Ultra-small carbon dots boost bioelectricity generation by accelerating extracellular electron transfer. J. Power Sources 2024, 610, 234711 10.1016/j.jpowsour.2024.234711.

[ref19] Liu L.; Choi S. Enhanced biophotoelectricity generation in cyanobacterial biophotovoltaics with intracellularly biosynthesized gold nanoparticles. J. Power Sources 2021, 506, 230251 10.1016/j.jpowsour.2021.230251.

[ref20] Kim Y.; Smith J. G.; Jain P. K. Harvesting multiple electron–hole pairs generated through plasmonic excitation of Au nanoparticles. Nat. Chem. 2018, 10, 763–769. 10.1038/s41557-018-0054-3. 29736005

[ref21] Wang X.-N.; Niu M.-T.; Fan J.-X.; Chen Q.-W.; Zhang X.-Z. Photoelectric Bacteria Enhance the In Situ Production of Tetrodotoxin for Antitumor Therapy. Nano Lett. 2021, 21, 4270–4279. 10.1021/acs.nanolett.1c00408. 33955768

[ref22] Italiano F.; Agostiano A.; Belviso B. D.; Caliandro R.; Carrozzini B.; Comparelli R.; Melillo M. T.; Mesto E.; Tempesta G.; Trotta M. Interaction between the photosynthetic anoxygenic microorganism Rhodobacter sphaeroides and soluble gold compounds. From toxicity to gold nanoparticle synthesis. Colloids Surf., B 2018, 172, 362–371. 10.1016/j.colsurfb.2018.06.010. 30189387

[ref23] Okoro G.; Husain S.; Saukani M.; Mutalik C.; Yougbaré S.; Hsiao Y.-C.; Kuo T.-R. Emerging Trends in Nanomaterials for Photosynthetic Biohybrid Systems. ACS Mater. Lett. 2023, 5, 95–115. 10.1021/acsmaterialslett.2c00752.

[ref24] Piella J.; Bastús N. G.; Puntes V. Size-Controlled Synthesis of Sub-10-nanometer Citrate-Stabilized Gold Nanoparticles and Related Optical Properties. Chem. Mater. 2016, 28, 1066–1075. 10.1021/acs.chemmater.5b04406.

[ref25] Draviana H. T.; Fitriannisa I.; Khafid M.; Krisnawati D. I.; Widodo Lai W.; Lai C.-H.; Fan Y.-J.; Kuo T.-R. Size and charge effects of metal nanoclusters on antibacterial mechanisms. J. Nanobiotechnol. 2023, 21, 428 10.1186/s12951-023-02208-3. PMC10648733 37968705

[ref26] Kuo J.-C.; Tan S.-H.; Hsiao Y.-C.; Mutalik C.; Chen H.-M.; Yougbaré S.; Kuo T.-R. Unveiling the Antibacterial Mechanism of Gold Nanoclusters via In Situ Transmission Electron Microscopy. ACS Sustainable Chem. Eng. 2022, 10, 464–471. 10.1021/acssuschemeng.1c06714.

[ref27] Mutalik C.; Lin I. H.; Krisnawati D. I.; Khaerunnisa S.; Khafid M.; Widodo H.; Hsiao Y.; Kuo T. R. Antibacterial Pathways in Transition Metal-Based Nanocomposites: A Mechanistic Overview. Int. J. Nanomed. 2022, 17, 6821–6842. 10.2147/IJN.S392081. PMC9809169 36605560

[ref28] Dolai J.; Mandal K.; Jana N. R. Nanoparticle Size Effects in Biomedical Applications. ACS Appl. Nano Mater. 2021, 4, 6471–6496. 10.1021/acsanm.1c00987.

[ref29] Torquato L. D. D. M.; Grattieri M. Photobioelectrochemistry of intact photosynthetic bacteria: Advances and future outlook. Curr. Opin. Electrochem. 2022, 34, 101018 10.1016/j.coelec.2022.101018.

[ref30] Vasiliadou I. A.; Berná A.; Manchon C.; Melero J. A.; Martinez F.; Esteve-Nuñez A.; Puyol D. Biological and Bioelectrochemical Systems for Hydrogen Production and Carbon Fixation Using Purple Phototrophic Bacteria. Front. Energy Res. 2018, 6, 107 10.3389/fenrg.2018.00107.

[ref31] Corona V. M.; Le Borgne S.; Revah S.; Morales M. Effect of light-dark cycles on hydrogen and poly-β-hydroxybutyrate production by a photoheterotrophic culture and Rhodobacter capsulatus using a dark fermentation effluent as substrate. Bioresour. Technol. 2017, 226, 238–246. 10.1016/j.biortech.2016.12.021. 28011238

[ref32] Gupta A.; Landis R. F.; Rotello V. M. Nanoparticle-Based Antimicrobials: Surface Functionality is Critical. F1000res 2016, 5, 364 10.12688/f1000research.7595.1. PMC4798155 27006760

[ref33] Turkevich J.; Stevenson P. C.; Hillier J. A study of the nucleation and growth processes in the synthesis of colloidal gold. Discuss. Faraday Soc. 1951, 11, 55–75. 10.1039/df9511100055.

[ref34] Duff D. G.; Baiker A.; Edwards P. P. A new hydrosol of gold clusters. 1. Formation and particle size variation. Langmuir 1993, 9, 2301–2309. 10.1021/la00033a010.

[ref35] Sharma B.; Rabinal M. K. Biologically active l-cysteine as a reducing/capping agent for controlled tuning of gold nanoparticles. J. Alloys Compd. 2015, 649, 11–18. 10.1016/j.jallcom.2015.06.160.

[ref36] Rosen J. E.; Gu F. X. Surface functionalization of silica nanoparticles with cysteine: a low-fouling zwitterionic surface. Langmuir 2011, 27, 10507–10513. 10.1021/la201940r. 21761888

[ref37] Leiske M. N.; De Geest B. G.; Hoogenboom R. Impact of the polymer backbone chemistry on interactions of amino-acid-derived zwitterionic polymers with cells. Bioact. Mater. 2023, 24, 524–534. 10.1016/j.bioactmat.2023.01.005. 36714331 PMC9860433

[ref38] Encinas N.; Angulo M.; Astorga C.; Colilla M.; Izquierdo-Barba I.; Vallet-Regí M. Mixed-charge pseudo-zwitterionic mesoporous silica nanoparticles with low-fouling and reduced cell uptake properties. Acta Biomater. 2019, 84, 317–327. 10.1016/j.actbio.2018.12.012. 30529082 PMC6718287

[ref39] Hu F.; Chen K.; Xu H.; Gu H. Design and preparation of bi-functionalized short-chain modified zwitterionic nanoparticles. Acta Biomater. 2018, 72, 239–247. 10.1016/j.actbio.2018.03.038. 29597022

[ref40] Bevilacqua P.; Nuzzo S.; Torino E.; Condorelli G.; Salvatore M.; Grimaldi A. M. Antifouling Strategies of Nanoparticles for Diagnostic and Therapeutic Application: A Systematic Review of the Literature. Nanomaterials 2021, 11, 780 10.3390/nano11030780. 33803884 PMC8003124

[ref41] Mateo J. M.; Hoz A. D. L.; Usón L.; Arruebo M.; Sebastian V.; Gomez M. V. Insights into the mechanism of the formation of noble metal nanoparticles by in situ NMR spectroscopy. Nanoscale Adv. 2020, 2, 3954–3962. 10.1039/D0NA00159G. 36132804 PMC9417889

[ref42] Philip D. Synthesis and spectroscopic characterization of gold nanoparticles. Spectrochim. Acta, Part A 2008, 71, 80–85. 10.1016/j.saa.2007.11.012. 18155956

[ref43] Amendola V.; Meneghetti M. Size Evaluation of Gold Nanoparticles by UV–vis Spectroscopy. J. Phys. Chem. C 2009, 113, 4277–4285. 10.1021/jp8082425.

[ref44] Hueso J. L.; Sebastián V.; Mayoral Á.; Usón L.; Arruebo M.; Santamaría J. Beyond gold: rediscovering tetrakis-(hydroxymethyl)-phosphonium chloride (THPC) as an effective agent for the synthesis of ultra-small noble metal nanoparticles and Pt-containing nanoalloys. RSC Adv. 2013, 3, 10427–10433. 10.1039/c3ra40774h.

[ref45] Pham T.; Jackson J. B.; Halas N. J.; Lee T. R. Preparation and Characterization of Gold Nanoshells Coated with Self-Assembled Monolayers. Langmuir 2002, 18, 4915–4920. 10.1021/la015561y.

[ref46] Jürgensen A.; Raschke H.; Esser N.; Hergenröder R. An in situ XPS study of L-cysteine co-adsorbed with water on polycrystalline copper and gold. Appl. Surf. Sci. 2018, 435, 870–879. 10.1016/j.apsusc.2017.11.150.

[ref47] Wu R.; Cui L.; Chen L.; Wang C.; Cao C.; Sheng G.; Yu H.; Zhao F. Effects of Bio-Au Nanoparticles on Electrochemical Activity of Shewanella oneidensis Wild Type and ΔomcA/mtrC Mutant. Sci. Rep. 2013, 3 (1), 3307 10.1038/srep03307. 24264440 PMC3837306

[ref48] Wu H.; Tito N.; Giraldo J. P. Anionic Cerium Oxide Nanoparticles Protect Plant Photosynthesis from Abiotic Stress by Scavenging Reactive Oxygen Species. ACS Nano 2017, 11, 11283–11297. 10.1021/acsnano.7b05723. 29099581

[ref49] Beaver K.; Gaffney E. M.; Minteer S. D. Understanding metabolic bioelectrocatalysis of the purple bacterium Rhodobacter capsulatus through substrate modulation. Electrochim. Acta 2022, 416, 140291 10.1016/j.electacta.2022.140291.

[ref50] Grattieri M.; Labarile R.; Buscemi G.; Trotta M. The periodic table of photosynthetic purple non-sulfur bacteria: intact cell-metal ions interactions. Photochem. Photobiol. Sci 2022, 21, 101–111. 10.1007/s43630-021-00116-9. 34748197

[ref51] Grattieri M.; Rhodes Z.; Hickey D. P.; Beaver K.; Minteer S. D. Understanding Biophotocurrent Generation in Photosynthetic Purple Bacteria. ACS Catal. 2019, 9, 867–873. 10.1021/acscatal.8b04464.

[ref52] Hasan K.; Patil S. A.; Gorecki K.; Leech D.; Hägerhäll C.; Gorton L. Electrochemical communication between heterotrophically grown Rhodobacter capsulatus with electrodes mediated by an osmium redox polymer. Bioelectrochemistry 2013, 93, 30–36. 10.1016/j.bioelechem.2012.05.004. 22749669

[ref53] Grattieri M.; Patterson S.; Copeland J.; Klunder K.; Minteer S. D. Purple Bacteria and 3D Redox Hydrogels for Bioinspired Photo-bioelectrocatalysis. ChemSuschem 2020, 13, 230–237. 10.1002/cssc.201902116. 31600418

[ref54] Grattieri M. Purple bacteria photo-bioelectrochemistry: enthralling challenges and opportunities. Photochem. Photobiol. Sci. 2020, 19, 424–435. 10.1039/c9pp00470j. 32052814

[ref55] Wenzel T.; Härtter D.; Bombelli P.; Howe C. J.; Steiner U. Porous translucent electrodes enhance current generation from photosynthetic biofilms. Nat. Commun. 2018, 9 (1), 1299 10.1038/s41467-018-03320-x. 29610519 PMC5880806

