# Supplementary material for: Deciphering the Role of Inorganic Nanoparticles’ Surface Functionalization on Biohybrid Microbial Photoelectrodes
Source: ACS Appl Mater Interfaces. 2024 Oct 20;16(43):58598–608. doi: 10.1021/acsami.4c12070 (PMC11533150; doi:10.1021/acsami.4c12070)
Supplement: Supplementary file 1 — am4c12070_si_001.pdf [file am4c12070_si_001.pdf]

Supporting Information

Deciphering the Role of Inorganic Nanoparticles' Surface Functionalization on Biohybrid Microbial Photoelectrodes

Pierluigi Lasala[1,2], Rosa Maria Matteucci[2, 3], Saverio Roberto Volpicella[1], Jefferson Honorio Franco[1], Doriana Debellis[4], Federico Catalano[4], Antonella Milella[1], Roberto Grisorio[5], Gian Paolo Suranna [5,6], Angela Agostiano[1,2], Maria Lucia Curri[1,2,7], Elisabetta Fanizza[1,2,7*] and Matteo Grattieri[1,2,7*]

[1] *Department of Chemistry, University of Bari, Via Orabona 4, Bari 70125, Italy*

[2] *CNR-IPCF, SS Bari, Via Orabona 4, Bari 70125, Italy*

[3] *Polytechnic University of Bari, Via Orabona 4, Bari 70125, Italy*

[4] *Electron Microscopy Facility, Istituto Italiano di Tecnologia, Via Morego, 30, Genoa 16163, Italy*

[5] *Dipartimento di Ingegneria Civile, Ambientale, del Territorio, Edile e di Chimica (DICATECh), Politecnico di Bari, Via Orabona 4, Bari 70125, Italy*

[6] *CNR-NANOTEC, Institute of Nanotechnology, c/o Campus Ecoteckne, Via Monteroni, Lecce 73100, Italy*

[7] *Consorzio Interuniversitario Nazionale per la Scienza e Tecnologia dei Materiali (INSTM), Bari Research Unit, Via Orabona 4, Bari 70125, Italy*

*elisabetta.fanizza@uniba.it; matteo.grattieri@uniba.it

TABLE OF CONTENTS:

Figure S1.	DLS, FT-IR and thermogravimetric analyses.	S-3
Figure S2.	UV-Vis absorption spectra of colloidal solutions.	S-3
Figure S3.	XPS high resolution and 1H-NMR spectra.	S-4
Figure S4.	Spectroscopic and morphologic characterization of Au@MPA NPs.	S-4
Figure S5.	Spectroscopic and morphologic characterization of Au@CysAm NPs.	S-5
Figure S6.	Time evolution of the normalized optical density for bacterial growths.	S-5
Figure S7.	UV-Vis-NIR absorption spectra and size distribution analysis of wild type and modified bacteria exposed to NPs.	S-6
Figure S8.	Energy dispersive X-ray spectroscopy of wild type and modified bacteria exposed to NPs.	S-6
Figure S9.	Cyclic voltammograms for control electrodes.	S-7
References		S-7

Figure S1 reports the dynamic light scattering characterization (A-C) of all the samples and the FT-IR in ATR mode (D-E) and the thermogravimetric characterization (F-H, f-h) of the Au@THPO and Au@Cys samples.

The number-weighted distribution (Figure S1B) of the hydrodynamic diameter (D_H) shows a monomodal distribution, with peak maximum (Figure S1A) quite well matching the NPs diameter as measured by TEM analysis.[1] FT-IR spectra of Au@THPO and Au@Cys have been recorded and compared with literature reported FT-IR spectra of Cys[2] and THPC[3, 4] (together with datasheet provided by several production companies) for FT-IR peak assignments. As confirmed by XPS and ^{1}H-NMR characterization - which indicated that the surface of Au@THPO primarily consists of THPO while Au@Cys contains THPC, THPO and Cys as ligands - the comparison of the two FT—IR spectra reveals the following: (i) a broad band at 1359 cm^{-1} in Au@THPO, ascribed to convolution of vibrational modes of different functional groups, CH_2 scissoring, O-H bending, and stretching of C-O and P-CH_2, splits into two distinct bands in Au@Cys. These peaks correspond to symmetrical stretching $C(=O)_2$ and O-H bending and P-CH_2 stretching at 1420 cm^{-1} and C-H bending of tertiary C-H group of branched cysteine structure at 1357 cm^{-1}; (ii) the stretching mode characteristic of the C-N bond is present in Au@Cys at 1240 cm^{-1}; and (iii) the S-H bond observable in cysteine, is absent in the Au@Cys NPs spectrum due to the thiol binding the NPs via S-Au bond.

To determine the organic content in Au NPs samples, we performed thermogravimetic analysis on both Au@THPO and Au@Cys. This analysis, using the first derivative curve, helps identify the temperatures at which weight losses occur and allows to qualitatively attribute these losses to specific moieties or unbound molecules. The thermogram of Au@THPO (Figure S1 F) shows a gradual continuous loss of nearly 10% from 75°C to 520°C, with distinct weight losses observed before 100°C and at 162°C, 231°C, 323°C, 420°C, 501°C (refer to the first derivative panel f). These losses are attributed to adsorbed water and different THPO moieties and fragments, respectively. Conversely, the thermogram of Au@Cys and its first derivative display some differences. The noticeable weight loss of about 4% at 153°C can be ascribed to moieties arising from Cys. Unlike Cys powder, characterized by small % of weight loss at 153°C and a larger % of weight loss 222°C, the Cys bound to Au NPs appears to follow a different sublimation pathway, probably due to the strong covalent bonding of thiol groups to gold. For temperature above 200°C, the thermogram indicates a 6% weight loss at temperature similar to those observed for Au@THPO. This suggests that approximately 10% of the total weight of a Au@Cys sample is made up of the organic shell, with 4% attributable to Cys and the remaining 6% to THPC and THPO.

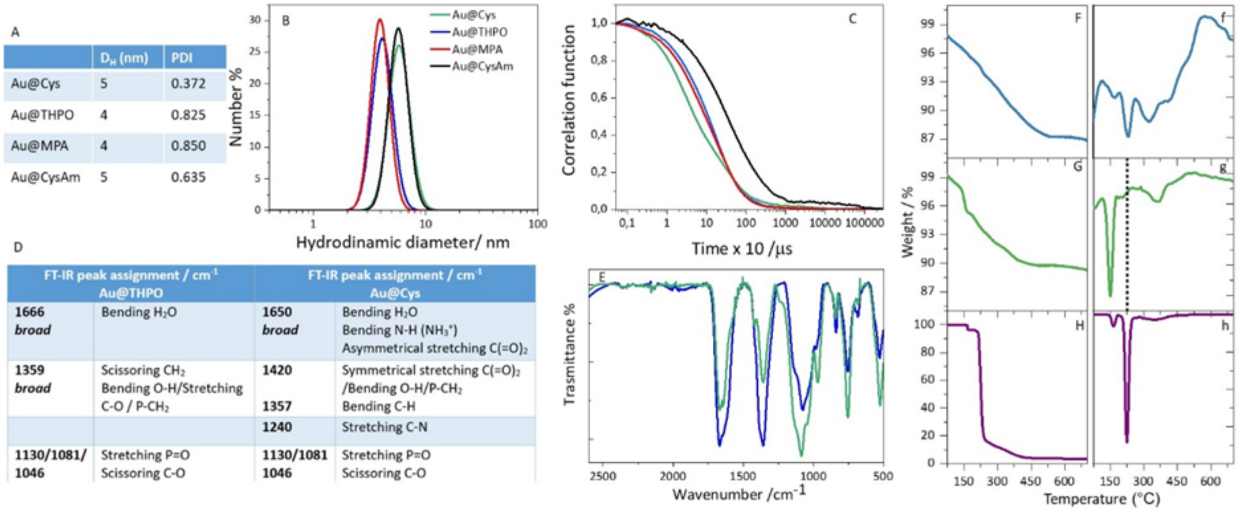

	D_H (nm)	PDI
Au@Cys	5	0.372
Au@THPO	4	0.825
Au@MPA	4	0.850
Au@CysAm	5	0.635

FT-IR peak assignment / cm^{-1} Au@THPO		FT-IR peak assignment / cm^{-1} Au@Cys	
**1666** ***broad***	Bending H_2O	**1650** ***broad***	Bending H_2O<br>Bending N-H (NH_3^+)<br>Asymmetrical stretching $C(=O)_2$
**1359** ***broad***	Scissoring CH_2<br>Bending O-H/Stretching C-O / P-CH_2	**1420**<br><br>**1357**	Symmetrical stretching $C(=O)_2$ /Bending O-H/P-CH_2<br>Bending C-H
		**1240**	Stretching C-N
**1130/1081/ 1046**	Stretching P=O<br>Scissoring C-O	**1130/1081 1046**	Stretching P=O<br>Scissoring C-O

Figure S1. (A-C) Dynamic light scattering measurements: table reporting hydrodynamic diameter (D_H) values and polydispersity index (PDI) for all the samples (A), plots of the number-weighted distribution (B) and correlation function (C) color code: Au@Cys green line, Au@THPO blue line, Au@MPA red line, Au@CysAm black line; (D-E) FT-IR in attenuated total reflection mode: table (D) reporting the characteristic stretching, bending and scissoring modes revealed by Au@THPO (blue line) and Au@Cys (green line) spectra reported in panel E; (F-H, f-h) thermogravimetric analysis carried out under nitrogen atmosphere in the 75°C-700 °C temperature range (F-H) and first derivative plot (f-h) of Au@THPO (blue line), Au@Cys (green line) and cysteine (magenta).

Time evolution of Au@Cys NPs absorption spectrum after Cys injection, overnight reaction, and purification has been compared with that of Au@THPO NPs, recorded at the same reaction times (Figure S2). The spectroscopic characterization highlights that at the early stage of the reaction, the absorption spectrum of Au@Cys NPs is only dominated by the scattering while the LSPR characteristic of small plasmonic Au NPs appears only at prolonged reaction time, being retained after purification. Conversely, a more pronounced LSPR band is shown by Au@THPO NPs.

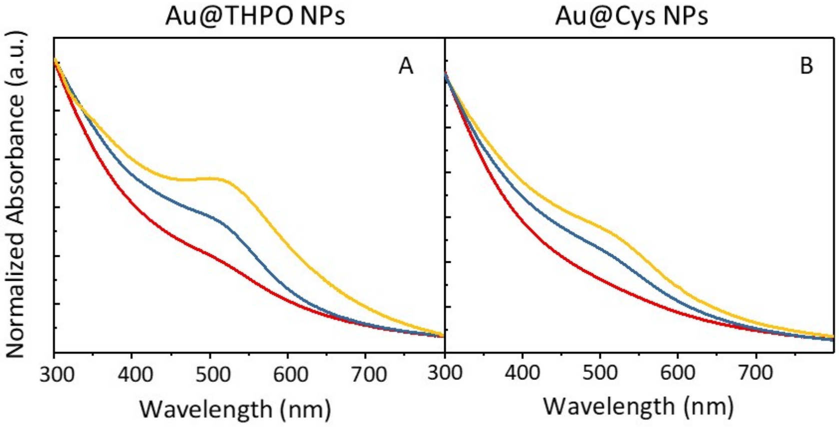

Figure S2. UV-Vis absorption spectrum of colloidal solution of Au@THPO (A) and Au@Cys (B) NPs recorded 10 minutes after Cys addition (red line), after 16 hours of reaction (blue line, overnight), and after purification (yellow line).

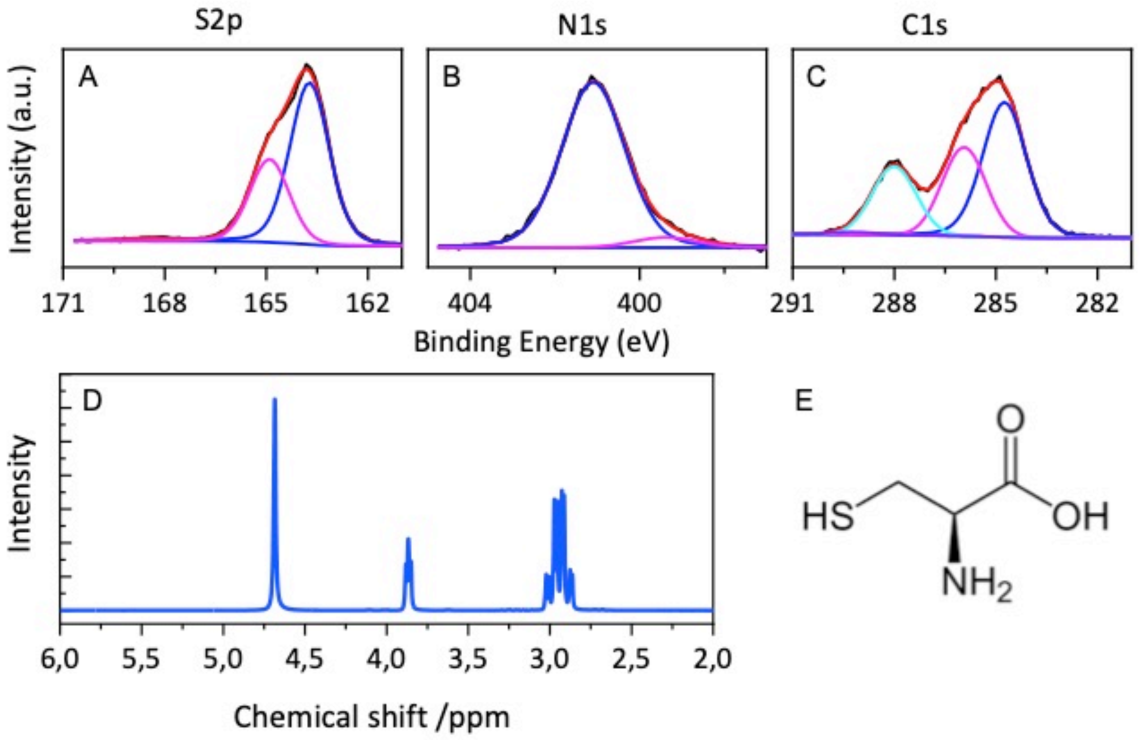

Figure S3. XPS high resolution spectra of S2p (A) N1s (B), C1s (C) and ^{1}H -NMR spectra (D) of cysteine. Molecular structure in panel E.

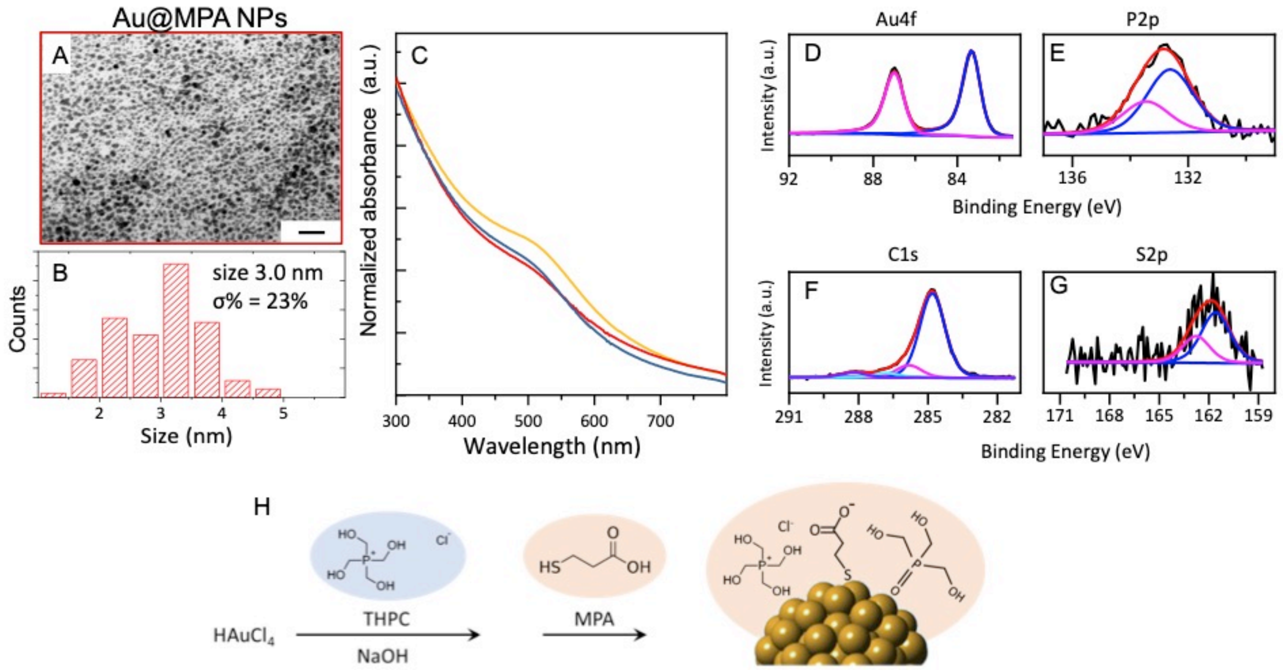

Figure S4. (A) TEM micrograph (scale bar 20 nm), (B) statistical analysis and size distribution, (C) time evolution of the UV-Vis absorption spectrum of Au NPs right after addition of 3 mercaptopropionic (MPA) (red line), overnight reaction (blue line), and purification (yellow line). XPS characterization (D-G) with high resolution spectra of Au4f (D), P2p (E), C1s (F) and N1s (G) for Au@MPA NPs and (H) sketch of the synthetic path and surface functionalization

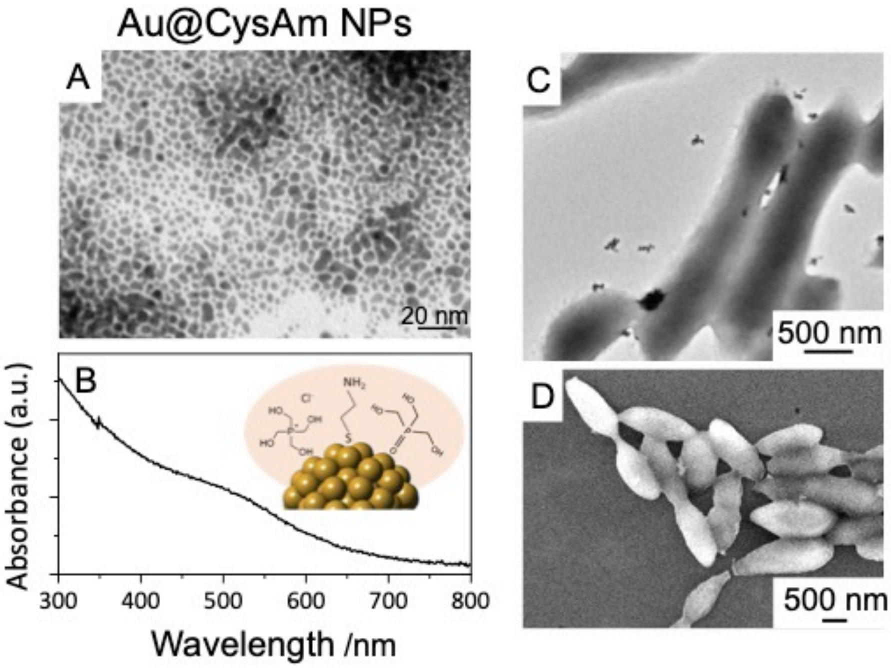

Figures S5. (A) TEM micrograph, (B) UV-vis absorption spectrum of Au NPs@CysAm, inset: sketch of the Au@CysAm NPs, (C) TEM, and (D) SEM micrograph of *R.capsulatus*/Au@CysAm NPs.

In Figure S6 the optical density (OD) of *R. capsulatus* incubated with different concentration of Au@Cys NPs is reported over a time of 50 hours. NPs loading set at a concentration of 50 μg·mL^{-1} was selected as condition offering the high NP concentration while avoiding significant alteration bacterial cells vitality and growth kinetics.

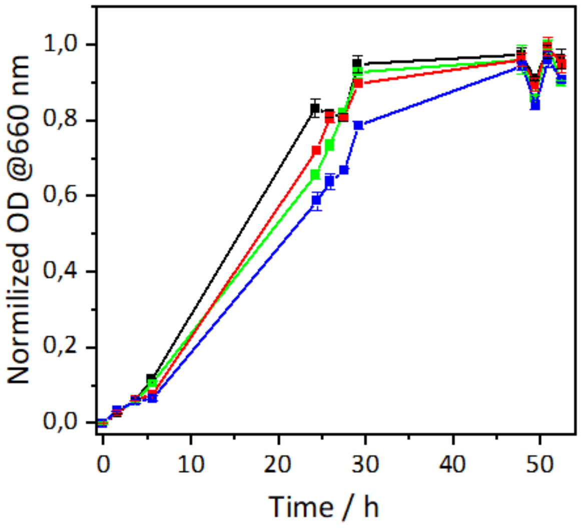

Figure S6. Time evolution of the normalized optical density measured at 660 nm of *R.capsulatus* (black) and *R.capsulatus*/Au@Cys NPs, with Au@Cys NPs loaded at different concentration 10μg mL^{-1} (green line and symbol), 50μg mL^{-1} (red) and 100μg mL^{-1} (blue).

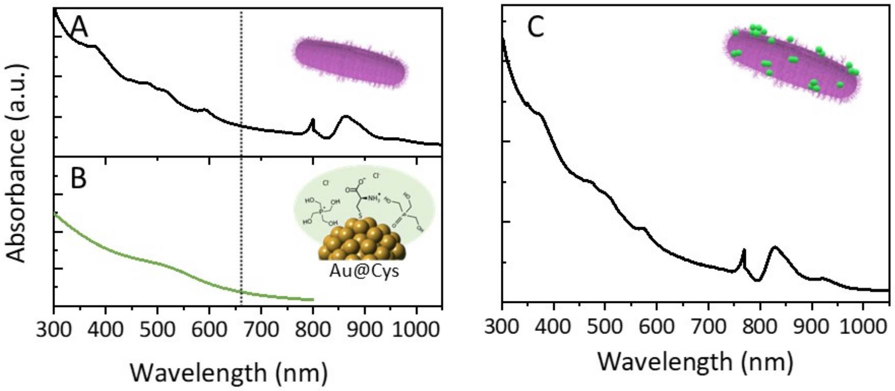

D

	R.caps	*R.Caps*/Au@Cys NPs	*R.Caps*/Au@THPO NPs	*R.Caps*/Au@MPA NPs	*R.Caps*/Au@CysAm NPs
Length (µm)	1.42 ± 0.19	1.32 ± 0.12	1.22 ± 0.13	1.81 ± 0.33	1. 80 ± 0.30
Width (µm)	0.58 ± 0.07	0.55 ± 0.05	0.52 ± 0.06	0.58 ± 0.06	0.56 ± 0.08
Aspect ratio	2.45 ± 0.60	2.40± 0.40	2.35 ± 0.50	3.12 ± 0.90	3,21± 0.99

Figure S7. (A) UV-Vis-NIR absorption spectra of *R. capsulatus*, (B) Au@Cys NPs and (C) *R.capsulatus*/Au@Cys NPs. Table with size distributions of *R. capsulatu*s cells and *R. capsulatus* cells incubated with each NPs sample (D).

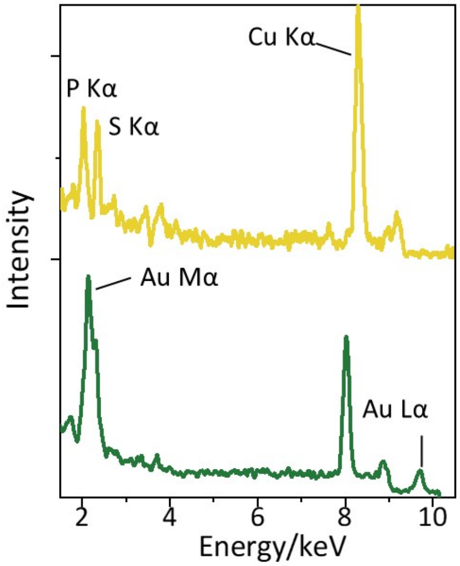

Figure S8. Energy dispersive X-ray spectroscopy of wild type *R. capsulatus* (yellow trace) and *R.capsulatus*/Au@Cys NPs biohybrids (green trace). Similar spectra are obtained for *R.capsulatus*/Au@THPCO NPs, *R.capsulatus*/Au@MPA NPs and *R.capsulatus*/Au@CysAm NPs biohybrids.

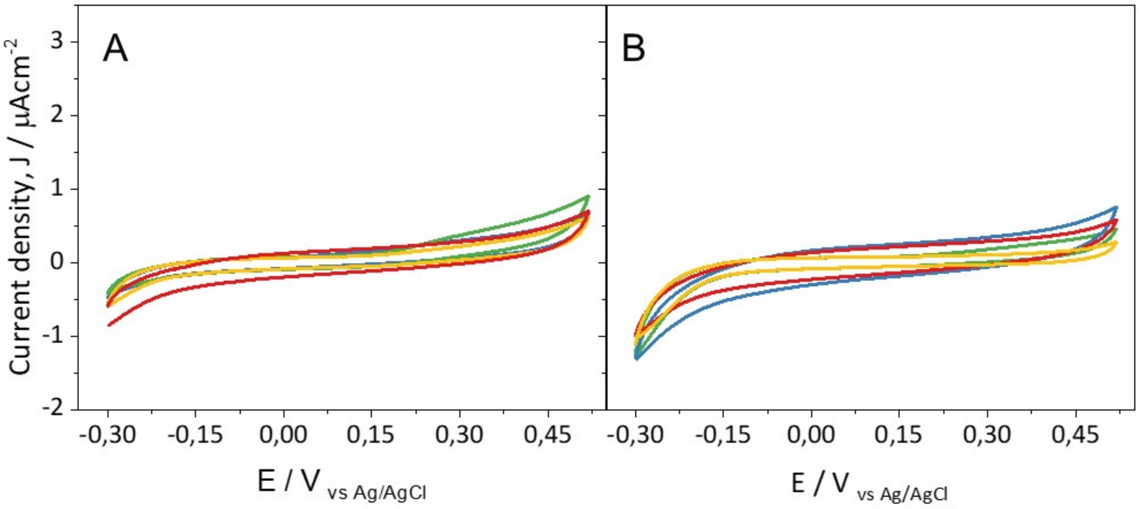

Figure S9. Cyclic voltammograms of heat-treated *R.capsulatus* (yellow trace), and heat-treated *R.capsulatus* after the incubation with Au@Cys NPs (green trace), Au@MPA NPs (red trace) and Au@THPO NPs (blue trace), under illumination (A) and in dark conditions (B). Scan rate: 2 mVs^{-1}.

REFERENCES

(1) Filippov, S. K.; Khusnutdinov, R.; Murmiliuk, A.; Inam, W.; Zakharova, L. Y.; Zhang, H.; Khutoryanskiy, V. V. Dynamic light scattering and transmission electron microscopy in drug delivery: a roadmap for correct characterization of nanoparticles and interpretation of results. *Materials Horizons* **2023**, *10*, 5354-5370.

(2) Devi, S.; Singh, B.; Paul, A. K.; Tyagi, S. Highly sensitive and selective detection of trinitrotoluene using cysteine-capped gold nanoparticles. *Analytical Methods* **2016**, *8*, 4398-4405.

(3) Zhao, B.; Kolibaba, T. J.; Lazar, S.; Grunlan, J. C. Environmentally-benign, water-based covalent polymer network for flame retardant cotton. *Cellulose* **2021**, *28*, 5855-5866.

(4) Bryan, W. W.; Jamison, A. C.; Chinwangso, P.; Rittikulsittichai, S.; Lee, T.-C.; Lee, T. R. Preparation of THPC-generated silver, platinum, and palladium nanoparticles and their use in the synthesis of Ag, Pt, Pd, and Pt/Ag nanoshells. *RSC Advances* **2016**, *6*, 68150-68159.
